# The quality of antiretroviral medicines: an uncertain problem

**DOI:** 10.1136/bmjgh-2022-011423

**Published:** 2023-03-15

**Authors:** Ngan Thi Do, Phonepasith Boupha, Paul N Newton, Céline Caillet

**Affiliations:** 1 Lao-Oxford-Mahosot Hospital-Wellcome Trust Research Unit, Medicine Quality Research Group, Microbiology Laboratory, Mahosot Hospital, Vientiane, Lao People's Democratic Republic; 2 Nuffield Department of Medicine, Infectious Diseases Data Observatory (IDDO)/WorldWide Antimalarial Resistance Network (WWARN), Medicine Quality Research Group, Oxford University Centre for Tropical Medicine and Global Health, Oxford, UK; 3 Nuffield Department of Medicine, Medicine Quality Research Group, Oxford University Centre for Tropical Medicine and Global Health, Oxford, UK

**Keywords:** Public Health, AIDS, HIV, Systematic review, Epidemiology

## Abstract

**Objectives:**

Substandard and falsified (SF) antiretrovirals (ARVs) risk poor outcomes and drug resistance, potentially affecting millions of people in need of treatment and prevention. We assessed the available evidence on SF ARV and related medical devices to discuss their potential public health impact.

**Methods:**

Searches were conducted in Embase, PubMed, Google, Google Scholar, Web of Science and websites with interest in ARV quality in English and French up to 30 November 2021. Publications reporting on the prevalence of SF ARV were assessed in a quantitative analysis using the Medicine Quality Assessment Reporting Guidelines (MEDQUARG).

**Results:**

We included 205 publications on SF ARV and 11 on SF medical devices. Nineteen prevalence surveys of SF ARV, published between 2003 and 2021, were included, with no surveys relevant to SF medical devices. The prevalence survey sample size ranged from 3 to 2630 samples (median (Q1–Q3): 16.0 (10.5–44.5); 3 (15.8%) used random outlet sampling methods. Of the 3713 samples included in the prevalence surveys, 1.4% (n=51) failed at least one test. Efavirenz, nevirapine and lamivudine-nevirapine-stavudine combination were the most surveyed ARV with failure frequencies of 3.6% (7/193), 2.6% (5/192) and 2.8% (5/177), respectively. The median (Q1%–Q3%) concordance with the MEDQUARG criteria was 42.3% (34.6%–55.8%).

**Conclusion:**

These results suggest that there are few data in the public domain of the quality of ARV in supply chains; the proportion of SF ARV is relatively low in comparison to other classes of essential medicines. Even a low proportion of the ARV supply chain being poor quality could make a large difference in the HIV/AIDS international landscape. The 95-95-95 target for 2026 and other international targets could be greatly hampered if even 1% of the millions of people taking ARV (for both prevention and prophylaxis) receive medicines that do not meet quality standards. More surveillance of SF ARV is needed to ensure issues are detected.

**WHAT IS ALREADY KNOWN ON THIS TOPIC**

- Substandard and falsified (SF) antiretrovirals (ARVs) lead to negative health impacts for patients with HIV infection, including poor patients outcomes and economic losses. They also likely to have global public health impact engendering drug resistance. However, data on SF ARV are scattered without global understanding of their epidemiology and impact.

**WHAT THIS STUDY ADDS**

- In the 19 studies, we identified that aimed to understand their epidemiology, 1.4% of the 3713 ARV samples failed at least one quality test.
- However, this estimate is not generalisable globally due to major gaps in the evidence, with geographical disparities and survey methodology issues. Prevalence surveys mainly included ARV samples collected in Africa and we found no publicly available evidence for almost 90% of national states.

**HOW THIS STUDY MIGHT AFFECT RESEARCH, PRACTICE OR POLICY**

- Our findings suggest that SF ARV are a public health issue as even a low proportion of the ARV supply chain being poor quality could make a large difference for the millions of patients who take them globally.
- More research with robust methodology and reporting is required to provide more precise estimates of the extent of the problem, where and what the problems are and the potential impact of SF ARV on drug resistance and patient outcome, to better inform interventions and policy.

## Introduction

Antiretrovirals (ARVs) are primarily used for the treatment and prevention of infection by the human immunodeficiency virus (HIV).^1^ According to the WHO, approximately 38.4 million people were living with HIV at the end of 2021^2^ and by July 2022, the HIV/AIDS had caused 40.1 million deaths globally.^2^ Approximately 850 children became infected with HIV and approximately 310 children died each day in 2021 from AIDS-related causes.^3^

Globally, 75% of HIV-infected people were receiving antiretroviral therapy (ART) at the end of 2021.^2 4^ With no cure or vaccine currently available, access to quality ART is crucial to control the infection and help prevent transmission. The WHO estimated that between 2000 and 2019 ARV saved 15.3 million lives and reduced the percentage of new HIV infections by 39% and HIV-related deaths by 51%.^5^

HIV drug resistance (HIVDR) affects the efficacy of ART, resulting in increased HIV-associated morbidity and mortality and transmission. According to surveys conducted in 10 countries in sub-Saharan Africa (2012–2020), nearly one-half of infants born to mothers infected with HIV presented with HIVDR to one or more non-nucleoside reverse transcriptase inhibitors (NNRTIs), one of the key classes of medicines for treatment and prevention of HIV transmission.^6 7^ Minimising the spread of HIVDR is critical to ensure long-term efficacy and durability of ARV.

The global ARV drugs market value exceeded US$ 24.7 billion in 2018.^8^ Projections suggest that it will be US$ 22.5 billion by 2024.

Substandard (due to within factory or supply chain errors) and falsified (due to fraud) (substandard and falsified, SF) medical products of all therapeutic classes have been found in many countries.^9 10^ The WHO estimated that around 10.5% of medical products are SF in L/MIC, with an estimated US%30.5 billion financial loss.^11^ A variety of defects have been found in SF medicines. They may contain one or several unexpected toxic active ingredients, too low or too high amounts of the expected active ingredients, they may contain none of the expected active ingredient(s) and they may also fail to dissolve properly, hence preventing the active ingredient(s) from reaching the blood stream, thus losing their efficacy. Hence, SF represent a serious public health problem. They also have a significant impact on clinical practice and the economy, and they generate loss of confidence in healthcare professionals and healthcare systems.^11^ Antibiotics and antimalarials are the most studied classes of medicines.^12–15^ A recent systematic review of the scientific literature showed that 17.4% of the 13 555 antibiotics tested for quality failed at least one quality test.^13^ In another systematic review, 15.4% of the 3414 medicines used for cardiovascular diseases failed at least one quality test.^15^ In both reviews, samples were mainly collected from low-income and middle-income countries and the number of samples tested per country was relatively small compared with the amount of medicines used globally. There is little scientific evidence publicly available on the quality of medicines available in high-income countries but the number and types of recalls by regulatory authorities show that these countries are not immune.^16–20^

Good quality ARVs are vital in the management of HIV infection and AIDS. The high number of people affected, the cost, the length of treatment and impaired access raise the risk of ARV falsification. Cases of SF ARV have been identified over the past decades and ARV are often quoted as medicines with common/recurring quality issues.^21–24^ However, as far as, we are aware there is no clear understanding on the epidemiology of SF ARV globally. This systematic review was conducted with the key objective to summarise the available evidence on ARV medicines quality globally, to discuss their potential impact for patients and society.

## Methods

### Search strategy

Search terms relevant to pharmaceutical quality (eg, ‘falsified’, ‘substandard’) were combined with search terms relevant to ARV and HIV/AIDS (online supplemental file 1). Systematic searches were conducted in Embase, PubMed, Google, Google Scholar and Web of Science in English and French up to 30 November 2021. The search terms were adapted for searches in MRA websites, and other websites with interest in medicines quality in English and French (online supplemental file 2). The articles from the first 20 pages of Google search results were screened for eligibility. Titles and abstracts were first screened and full texts of the identified articles were then assessed for eligibility. A manual search of the reference lists of the included articles was performed. Articles identified in previous systematic reviews by our group that included ARV medicines but not captured in our searches were also included.

10.1136/bmjgh-2022-011423.supp1 Supplementary data

10.1136/bmjgh-2022-011423.supp2 Supplementary data

### Eligibility criteria

Scientific articles and grey literature in English or French assessing or discussing the quality of ARV medicines were included. Articles containing scientific data on the prevalence of ARV medicines quality were the most relevant publications for this review. Other scientific articles included studies describing new tests or validation of innovative techniques to determine the quality of medicines in which ARV medicine samples were used to validate the technique, equivalence studies and quality control analyses. We also included reports of seizures, recalls, alerts by the MRAs or pharmaceutical companies and patients describing adverse reactions where the quality of the medicine was suspected to be the cause. The different types of study included in this review are described in online supplemental file 3.

10.1136/bmjgh-2022-011423.supp3 Supplementary data

We excluded data from publications describing the development/validation of analysis technique(s) for quality assessment of ARV medicines without sufficient information on the samples used and publications on the quality of herbal/mineral/animal part remedies claimed to treat HIV/AIDS.

We included medical devices for the diagnosis of HIV.

### Key definitions

Following the 2017 WHO definitions, falsified medicines are those that ‘deliberately/fraudulently misrepresent their identity, composition or source’.^25^ Substandard medicines are ‘authorised medical products that fail to meet either their quality standards or their specifications, or both’.^25^ This may result from negligence/errors during the manufacturing process or degradation through deterioration because of inappropriate storage/transport in the supply chain. There is inadequate evidence to distinguish poor quality medicines resulting from errors during the manufacturing process from subsequent degradation in the supply chain due to heat and humidity.

Pharmaceutical analysis relies on compendial tests described in pharmacopoeial monographs. For finished medicines, monographs commonly include the identification and quantification of Active Pharmaceutical Ingredient (API) content (using sophisticated standardised techniques such as liquid chromatography coupled with various detectors), dissolution testing, detection of specific levels of predetermined impurities/related substances, uniformity of dosage units and additional attributes depending on the formulation of the product (eg, tablet friability). In many studies included in this review, not all pharmacopoeial analyses were conducted and also a variety of non-pharmacopoeial assays were used, for example, for investigating specific contaminants or unstated APIs. Assay details were not always provided making it difficult to standardise the definition of a ‘failed sample’. Consequently, we define a failed sample as one for which at least one quality analysis test performed by the investigators gave a fail result, irrespective of the number and type of assays used.

As it is not possible to reliably classify a medicine as substandard or falsified without packaging analysis, products without packaging authentication that failed at least one quality test (ie, the results are outside the acceptable limits of the chosen specifications reference, either pharmacopoeia monograph or in-house specifications) are defined as ‘substandard or falsified’ (SorF).^14^ However, all samples that contained incorrect or no API were assumed to be falsified, although there is a (limited) risk of misclassification of such samples as falsified when they are substandard, due to gross manufacturing errors.

As in previous systematic reviews by our group,^13 15 26^ we define ‘failure frequency’ (FF) as the proportion of samples included in a prevalence survey that failed at least one quality test described in the report. We define a ‘data point’ as a specific location where medicines were collected for quality analysis, at a given time and for a given study. For medicines purchased online the location where the samples were received was extracted.

### Data collection

Data were manually extracted into the ‘Online Medicine Quality Data Manager’, an online data entry tool developed by the Infectious Diseases Data Observatory (IDDO) Informatics and the Lao-Oxford-Mahosot-Wellcome Trust Research Unit Medicine Quality team. Publication type (eg, report, original research article), year of publication, sampling type, location (country and city, where available) and type of outlet where samples were collected, the total number of samples collected, API/API combination name, number of samples failing medicine quality test(s), quality defect and the techniques that were used to analyse samples were entered in the online tool.

### Data analysis

FlySpeed SQL Query (V.3.5.4.2) was used to extract data from the online database and Microsoft Excel 2013 was used for data analysis. Qualitative variables were expressed as numbers and percentages (n (%)). Quantitative variables were expressed as median with first and third quartiles (Q1 and Q3, respectively).

### Quality of studies assessment: Medicine Quality Assessment Reporting Guidelines

The methodology and reporting of prevalence surveys were evaluated using the Medicine Quality Assessment Reporting Guidelines (MEDQUARG). MEDQUARG is a checklist of 26 items that should be included in reports of medicine quality surveys.^27^ All criteria had to be fulfilled for each item to be awarded one point. Prevalence surveys were assessed independently by two reviewers with a third person resolving any disagreement. Only the prevalence surveys published as original articles in scientific journals, following the Introduction/Methods/Results/Discussion section or similar style and published as reports or PhD thesis, were assessed.

This review was registered in the International Prospective Register for Systematic Review (PROSPERO, Registration No: CRD42016039531) and is reported according to the Preferred Reporting Items for Systematic Reviews and Meta-Analyses guidelines (online supplemental file 4).

10.1136/bmjgh-2022-011423.supp4 Supplementary data

### Patient and public involvement

Patients and/or the public were not involved in the design, or conduct, or reporting, or dissemination plans of this research.

## Results

### Overall literature on ARV medicines quality

**Figure 1:**
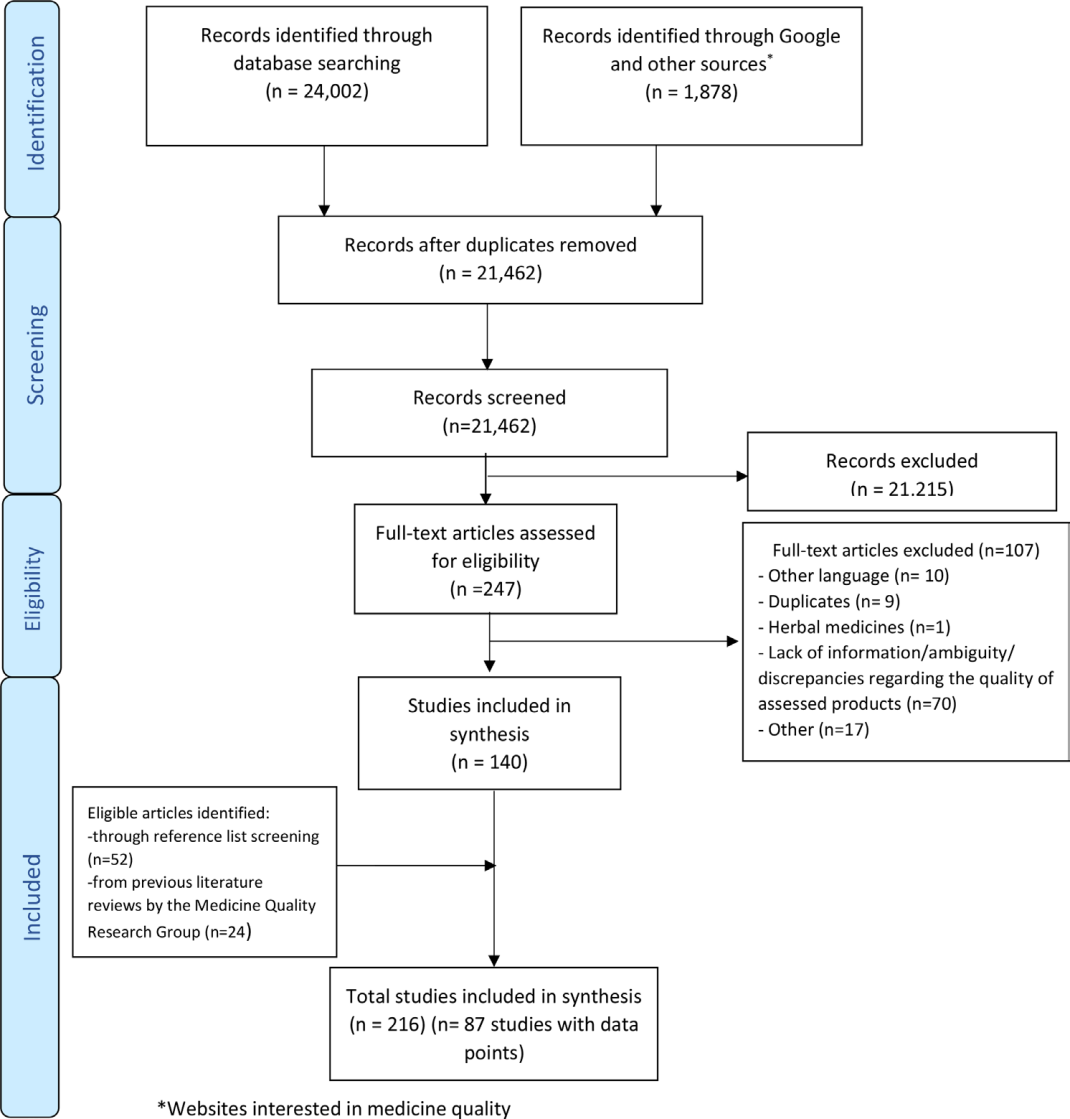
PRISMA flow chart of the selection process of the publications on antiretroviral medicines quality. PRISMA, Preferred Reporting Items for Systematic Reviews and Meta-Analyses.

After duplicates removal, 21 462 out of 25 880 publications gathered through electronic searches were screened by title and abstract (figure 1).

**Figure 2:**
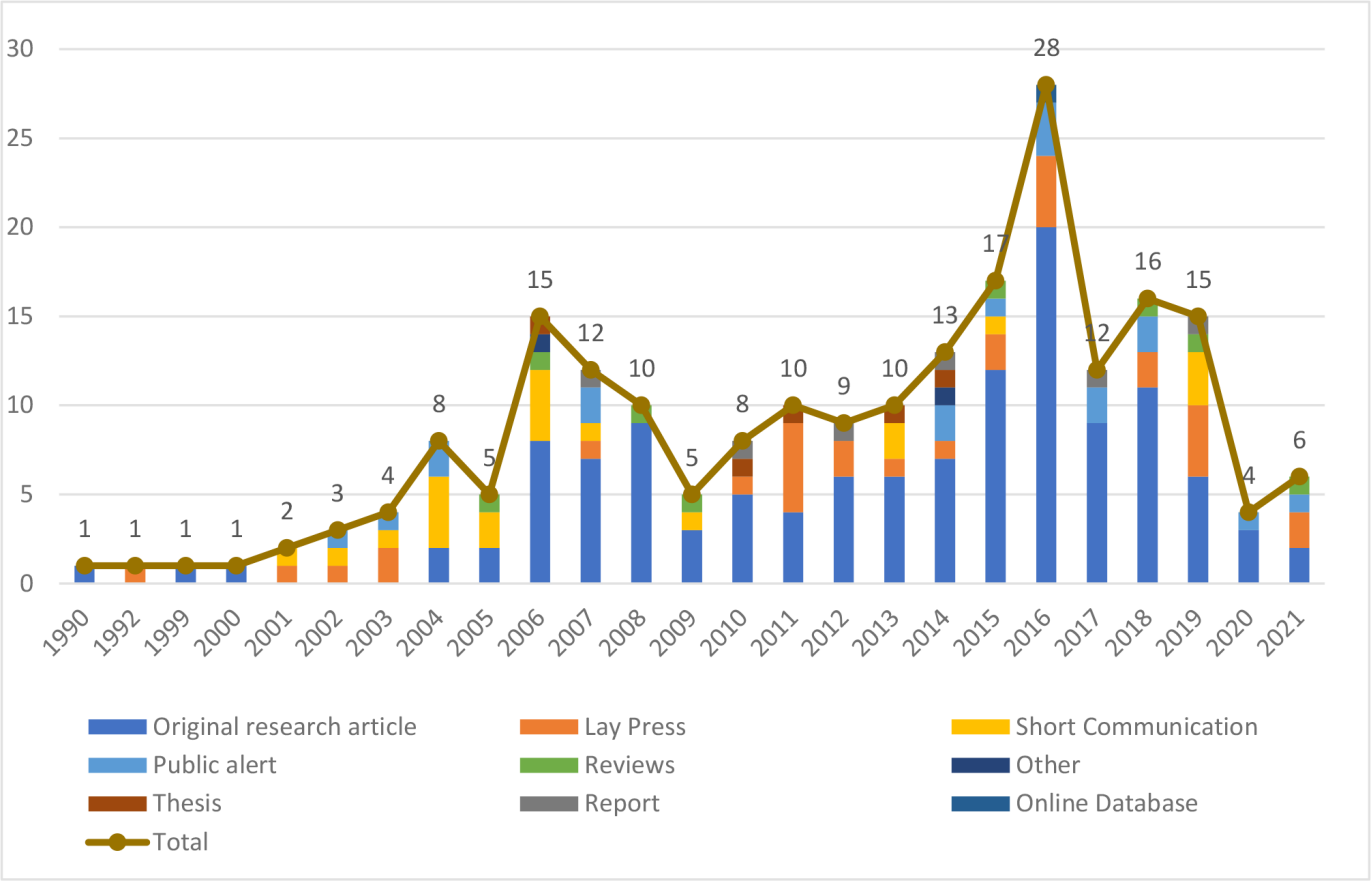
Number of publications per type and year of publication. (Note: publications published up to the 30 November 2021 only were included).

In total, 216 publications were included in this review, of which more than half were original research articles (57.9% (n=125)) and 13.9% (n=30) were lay press (figure 2). Most original research articles (89.6%, 112/125) were published in peer-reviewed journals. The number of publications related to ARV medicines quality per year was low between 1990 and 2003, reached a peak in 2016 (n=28 publications) and then decreased (figure 2).

Of the 216 publications, 205 were on ARV medicines quality and eleven on the quality of medical devices used in HIV. Of the 205 publications on ARV quality, 76 (37.1%) described the quality of ARV medicines in a specific location at a specific time with a total of 455 data points, and 129 (62.9%) did not contain data point information. No publication on medical devices for HIV diagnosis contained data on their quality in a specific location at a specific time. Out of 76 publications with data points, 19 (25.0%) were prevalence studies, 15 (19.7%) analytical technique development/validation, 8 (10.5%) routine quality control analysis, 4 (5.3%) equivalence studies, 1 (1.3%) bioavailability study and the data from the United States Pharmacopoeia's (USP) Medicines Quality Database were also included as one publication (1.3%) (online supplemental file 5). Others were recall/warning/alerts (n=16), seizures (n=7) and case reports (n=5) published in newspapers or medicines regulatory authorities websites.

10.1136/bmjgh-2022-011423.supp5 Supplementary data

A total of 4898 samples were collected and tested for quality, mainly in prevalence surveys (n=3713, 75.8%) and routine MRA quality control analysis (n=766, 15.6%). Of all samples, 59 (1.2%) failed at least one quality test. Of the failing samples, 54 (91.5%) were classified as SorF because no packaging analysis to assess the authenticity of the samples had been performed, 5 (8.5%) were substandard and no samples were classified as falsified.

All data are mapped and can be downloaded on the IDDO Medicine Quality Surveyor system (https://www.iddo.org/mqsurveyor/%23antiretrovirals).

### Prevalence surveys

Nineteen prevalence surveys published between 2003 and 2021 were included. Overall 3713 samples of 22 different APIs or combinations of APIs were collected in 21 countries (168 data points) on 4 continents. The sample size per study ranged from 3 to 2630 samples with a median (Q1–Q3) of 16.0 (10.5–44.5) samples per prevalence survey. The overall FF in prevalence surveys was 1.4% (51/3,713). Of the failing samples, 47 (92.2%) were classified as SorF, 4 (7.8%) were substandard and no samples were classified as falsified.

Three prevalence surveys used random sampling to select the outlets to be included (FF 2.1%, 9/419), 14 used convenience sampling only (FF 1.2%, 38/3,247), 1 used mixed random and convenience sampling designs (FF 0.0%, 0/42), and the sampling strategy was not described in one survey (FF 80.0%, 4/5) (online supplemental file 6).

10.1136/bmjgh-2022-011423.supp6 Supplementary data

We found no publicly available evidence for 174/195 (89.2%) of national states. About three-fourths (75.8%, n=2813/3713) of samples in prevalence surveys were collected from low-income countries, 18.7% (n=695/3,713) and 0.1% (n=37/3,713) were collected in middle-income and high-income countries, respectively (table 1). One hundred and sixty-eight samples (4.5%) were part of a large multicountry study but the FF were not broken down by country. Over 90% (3675/3713) of samples included in prevalence surveys were procured in Africa and Asia, representing 97.0% (3603/3713) and 1.9% (72/3713) of all the samples, respectively.

**Table 1:** Failure frequency by continent/country in prevalence surveys

Continent	Income	Country	No of publications	No of data points	Failure frequency % (n/N)
Americas					11.8 (2/17)
	HIC	USA	1	6	12.5 (2/16)
	UMIC	Jamaica	1	1	0.0 (0/1)
Europe					9.5 (2/21)
	HIC	Lithuania	2	5	40.0 (2/5)
	HIC	UK	1	3	0.0 (0/16)
Asia					2.8 (2/72)
	UMIC	China	1	3	33.3 (1/3)
	LMIC	Cambodia	1	1	14.3 (1/7)
	LMIC	India	1	7	0.0 (0/17)
	UMIC	Thailand	1	3	0.0 (0/3)
	Unknown	Unknown*	1	8	0.0 (0/42)
Africa					1.2 (45/3603)
	LIC	Ethiopia	1	4	25.0 (1/4)
	LMIC	Senegal	2	9	14.5 (8/55)
	UMIC	South Africa	3	9	9.1 (1/11)
	LMIC	Nigeria	2	11	5.7 (4/70)
	LIC	DR Congo	2	11	3.9 (2/51)
	LMIC	Zambia	5	17	3.1 (2/65)
	LMIC	Cameroon	2	11	1.4 (1/69)
	LIC	Tanzania	3	23	0.9 (24/2707)
	Unknown	Unknown†	1	1	0.8 (1/126)
	LMIC	Kenya	3	26	0.3 (1/394)
	LIC	Uganda	2	9	0.0 (0/51)
Total			19	168	1.4 (51/3713)

Because of the limited number of samples tested for quality in the studies included in this review, the figures should not be interpreted as representative of the prevalence of specific SF antiretroviral medicines (please refer to the discussion section of the current paper for more details).

*Multicountry study (Thailand and Vietnam) with no break down of the results by country.

†Multicountry study (Burkina Faso, Democratic Republic of the Congo, Nigeria, Rwanda and Zambia in Africa) with no break down of the results by country

DR Congo, Democratic Republic of the Congo; HIC, high-income country; LIC, low-income country; LMIC, lower-middle-income country; SF, substandard and falsified; UMIC, upper-middle-income country.

The FF was the highest in the Americas (11.8%, 2/17), followed by Europe (9.5%, 2/21), but the total number of samples tested was low. The FF was 1.2% (45/3603) in Africa and 2.8% (2/72) in Asia. The highest number of samples was collected in Tanzania (n=2707), with an FF of 0.9% (24/2707).

The proportion of samples of Efavirenz collected in prevalence surveys was the highest (5.2%, 193/3713) with FF=3.6% (7/193), followed by nevirapine (5.2%, 192/3713) with FF=2.6% (5/192) and lamivudine-nevirapine-stavudine combination (3.8%, 177/3713) with FF=2.8% (5/177), respectively (table 2).

**Table 2:** Failure frequency by API/API combination in prevalence survey

**API/API combination**	**No of publications**	**No of data points**	**Failure frequency % (n/N**)
Ritonavir	1	2	100.0 (2/2)
Indinavir	4	6	42.9 (6/14)
Lopinavir-ritonavir	4	5	18.2 (8/44)
Lamivudine-zidovudine-nevirapine	3	3	8.2 (7/85)
Stavudine	6	13	4.2 (4/96)
Efavirenz	10	23	3.6 (7/193)
Lamivudine-nevirapine-stavudine	7	14	2.8 (5/177)
Nevirapine	12	24	2.6 (5/192)
Zidovudine	7	18	1.9 (2/103)
Lamivudine	6	18	1.5 (2/132)
Lamivudine-zidovudine	5	11	1.5 (2/134)
Antiretroviral-unspecified	2	2	0.0 (1/2,325)
Abacavir	3	3	0.0 (0/33)
Abacavir-lamivudine	1	1	0.0 (0/1)
Amprenavir	1	1	0.0 (0/1)
Didanosin	4	4	0.0 (0/20)
Efavirenz-lamivudine-tenofovir disiproxil	1	1	0.0 (0/29)
Emtricitabine-efavirenz-tenofovir disiproxil	2	2	0.0 (0/28)
Emtricitabine-tenofovir disoproxil	2	4	0.0 (0/30)
Lamivudine-stavudine	4	5	0.0 (0/43)
Saquinavir	1	2	0.0 (0/2)
Tenofovir disoproxil	3	5	0.0 (0/25)
Tenofovir disoproxil-lamivudine	1	1	0.0 (0/3)
Total	19	168	1.4 (51/3713)

Because of the limited number of samples tested for quality in the studies included in this review, the figures should not be interpreted as representative of the prevalence of specific SF antiretroviral medicines (please refer to the discussion section of the current paper for more details).

API, active pharmaceutical ingredient; SF, substandard and falsified.

The FF of samples of ritonavir was the highest (100.0%, 2/2), followed by that of indinavir (42.9%, 6/14) but only few samples were tested.

Most of samples collected in prevalence surveys were tested for more than one quality attributes (93.8%, 3483/3713). Fourteen samples (1.4%, 14/1034) failed the API content test and 8 samples (1.3%, 8/616) failed the dissolution test. No sample (0.0%, 0/495) failed impurity/contaminant/related substances tests (online supplemental file 7).

10.1136/bmjgh-2022-011423.supp7 Supplementary data

Six samples out of 3256 (0.2 %) failed visual inspection of sample units (shape/colour uniformity, presence of contamination etc) and/or non-comparative packaging analysis (check of the availability of specific information and in some cases the conformity to packaging and labelling requirements with reference to MRA guidelines) in prevalence surveys. Of 14 samples that failed API content tests, 50.0% (7/14) contained lower API amount than stated, 42.9% (6/14) higher API amount and for 1 sample (7.1 %, 1/14) there was not enough information in the publication to determine whether it contained higher or lower amounts of API. Twelve out of 19 studies used High-Performance Liquid Chromatography (HPLC) methods (coupled with various detectors) for analysing API content (79.6%, 823/1034 samples).

The USP was the most commonly used (in 13/19 studies), followed by the British Pharmacopoeia and the International Pharmacopoeia (in 5 and 4 studies, respectively) (online supplemental file 6).

The highest FF was observed in samples collected from private pharmacies (28.0%, 7/25), followed by hospital/health centres (19.0%, 8/98), websites (7.7%, 2/26) and other government outlets (6.3%, 1/16) (online supplemental file 8). In total, 1302 samples were collected in multiple types of facilities with an FF of 2.2% (29/1302) but results of the quality tests were not given by outlet type. In additional, 2200 samples included in one study were collected in Tanzanian ports of entry with FF 0.0% (0/2200). For 21 samples, there was no information on the health facility where the samples were collected.

10.1136/bmjgh-2022-011423.supp8 Supplementary data

For the majority of the samples (93.3% (3464/3713)) included in prevalence surveys, there were no details on the stated manufacturer, or no breakdown of the samples by country of origin of the manufacturer (online supplemental file 9). The FF of the samples stated as made by Asian manufacturers (6.4%, 238/3713), was of 3.8% (9/238). The FF of samples stated as made by American manufacturers was the highest (14.3%, 1/7).

10.1136/bmjgh-2022-011423.supp9 Supplementary data

**Figure 3:**
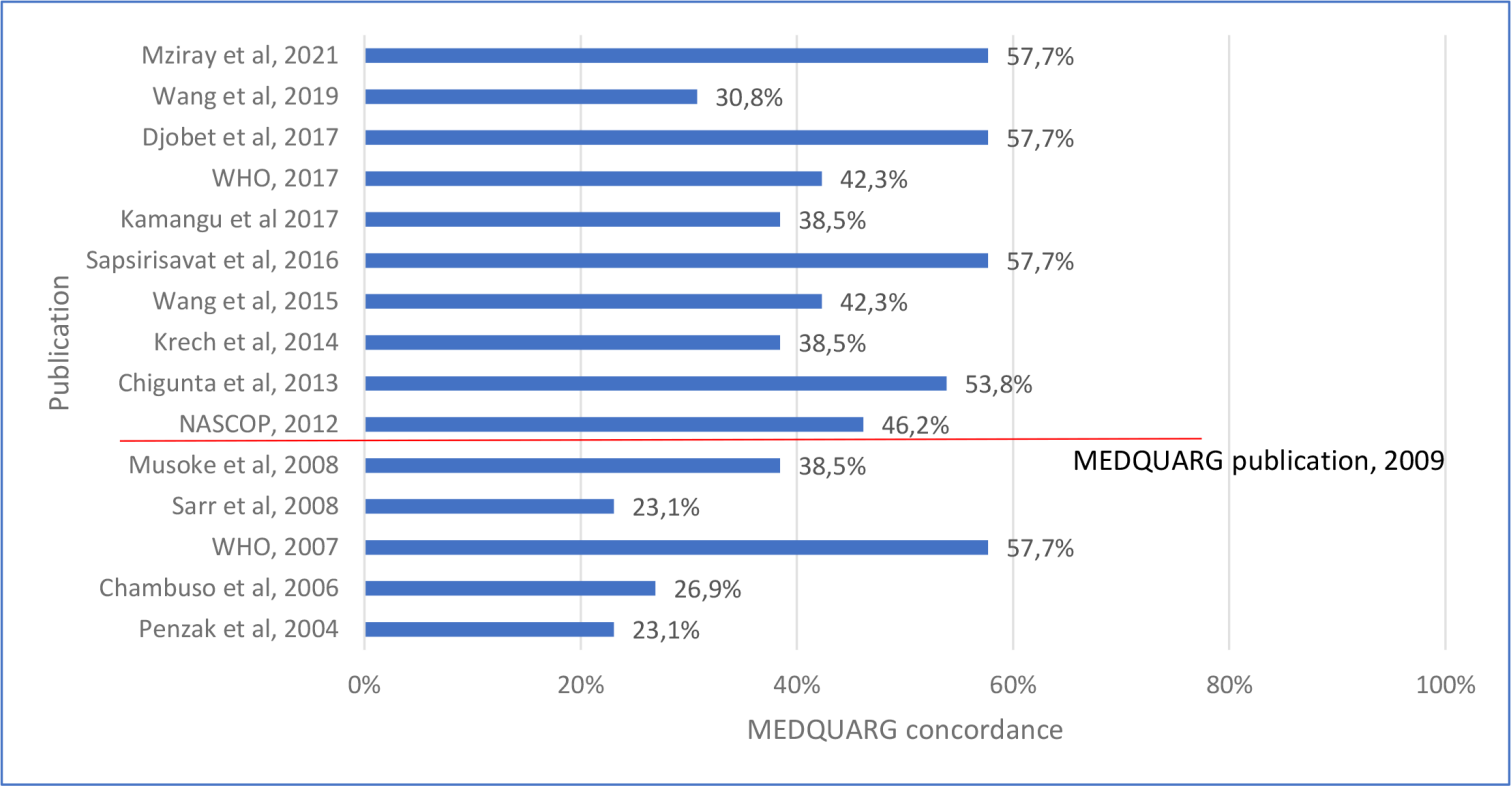
Percentage of concordance of the 15 prevalence surveys with the 26 items included in MEDQUARG checklist. MEDQUARG, Medicine Quality Assessment Reporting Guidelines.

The median (Q1%–Q3%) concordance with MEDQUARG items of 15 prevalence surveys that met the inclusion criteria for appraisal using MEDQUARG was 42.3% (34.6%–55.8%) (figure 3, online supplemental file 10).

10.1136/bmjgh-2022-011423.supp10 Supplementary data

### Quality of studies assessment

Although 10 surveys were reported after the publication of the MEDQUARG in 2009, none stated that the MEDQUARG guidelines were followed to report the findings. Three (20.0%) studies reported how the sample collectors presented to the seller (whether covert shopper, and what the sampler said/asked the seller) and 4 (26.7%) outlined the sampling design with sufficient details (online supplemental file 10). Only 40.0% (6/15) of the studies provided definitions on the quality of medicines or recognised the WHO definition. In 33.3% (5/15) of the surveys, the samples were clearly categorised as genuine, falsified or substandard or another equivalent terminology (or an explanation of the reason why this was not done); 33.3% (5/15) stated whether medicines were registered with the government in the location(s) sampled. Sixty per cent (10/15) of the studies reported with sufficient details the relationship between packaging and chemistry results. The MRA of the sampled country(ies) was either involved in the study (a representative of the MRA being an author in the paper) or was stated to be informed of its findings in four studies (26.7%).

### Seizures, recalls, case reports

Twenty-eight publications describing recalls/warning/alerts (n=16), seizures (n=7) and case reports (n=5) of SF ARV medicines were found during our searches (online supplemental file 11). Recalls of products of 14 APIs/combinations due to dissolution failure, API content or impurity/contaminant were found. In addition, 10 recalls/warning/alerts and seizures of HIV diagnostic test kit and HIV viral load for diagnostic test were identified (online supplemental file 12). Those include the substitution of 140 000 HIV rapid diagnostic test (RDT) kits by urinary pregnancy tests or resale of just-past expiry kits in India,^28^ and recall of one million of HIV testing kits in Kenya out of concern that they give false negative results.^29^

10.1136/bmjgh-2022-011423.supp11 Supplementary data

10.1136/bmjgh-2022-011423.supp12 Supplementary data

Other publications included in our review are listed in online supplemental file 13.

10.1136/bmjgh-2022-011423.supp13 Supplementary data

## Discussion

We synthesised the publicly available evidence on the quality of ARV medicines from different publicy accessible sources. Overall, 1.4% of 3713 ARV samples collected in 21 countries failed at least 1 quality test in the 19 prevalence studies. The limited sample sizes of the studies impede interpretation of the results. Drawing conclusions on the impact of SF ARV for patients and the community is also rendered difficult by the limited reporting of the findings in the various prevalence surveys, and often by the bias generated by their limited methodology, as described by others.^30 31^

The observed FF in this review is lower than the 4.2% (43/1,018) failure rate described in a recent review of the literature of studies conducted between 2007 and 2016 by the WHO.^11^ One recent study may result in underestimating the FF.^32^ In this study, from which more than half of the samples (2630 samples) described in the current review originated, 2200 samples collected at ports of entry in Tanzania over 4 years passed the Global Pharma Health Fund(GPHF)-Minilab initial screening tests, which included simple visual inspection of dosage units, API identification by thin-layer chromatography and disintegration tests. These 2200 samples were not further tested by reference testing in the laboratory. However, the same report describes that 10% samples of samples collected in other health structures that passed GPHF-Minilab screening were further tested using laboratory reference testing, resulting in an FF of 3%. Though the GPHF-Minilab has shown good performances to identify falsified samples containing none of the stated API, its sensitivity to identify substandard medicines containing lower or higher amounts of API is much lower.^33^ If the same 3% FF was applied to the 2200 samples collected in ports, the FF in this review would have been more than double (3.1% (117/3713)).

SF ‘HIV/hepatitis medicines’ represented 43/1500 (2.9%) of rapid alerts of reports to the Global Surveillance and Monitoring System between 2013 and 2017.^9^ Although ARVs are often quoted as one of the most affected products, together with other anti-infectives, the FF for ARV estimated here falls below that of other classes of medicines described in previous systematic reviews using the same methodology, such as for antibiotics (FF of 17.4% (2357/13 555)) and cardiovascular medicines (FF of 15.4% (525/3414)).^13 15^ In those reviews, samples were frequently procured in private sector’s facilities such as retail pharmacies, unlike in the current review in which an FF of 28.0% was observed in samples collected from private retail pharmacies, but only 25 samples were collected. ARV are often procured in LMIC within public or NGO vertical programmes which often follow stringent quality assurance systems and procure only WHO-prequalified medicines. However, in 2011 in Kenya nurses identified a falsified version of the ARV Zidolam-N, a WHO prequalified product, in Médecins Sans Frontières supplies relabelled fraudulently to extend its expiry date.^34^

The most common quality defects observed in prevalence surveys were lower or higher API content than stated on the label, failed dissolution tests (either too rapid or too slow), and impurity/contaminant/related substances tests. API in higher concentrations than expected risks not only poor outcomes to patients, but also lack of adherence through more frequent side effects. Using ARV medicines with too low API content and/or poor dissolution may lead to treatment failure, prolonged illness or death, and risks engendering the spread of drug resistant pathogens, although, as far as we are aware, the link between SF ARV and the emergence and spread of resistance has not been demonstrated.^35^

We found no publicly available evidence for almost 90% of national states, and for 17 of the 30 countries that bear 89% of the new HIV infections,^36^ which indicates an important lack of oversight of the risks. We found no study on the quality of dolutegravir, though this might be due to its only recent recommendation for use by the WHO (in combination with two NNRTIs) for newly diagnosed HIV patients.^37^ We also found limited information on tenofovir-based oral combinations recommended in 2015 by the WHO for pre-exposure prophylaxis (PrEP).^38^ An increasing number of countries are including self-testing of HIV in their national policies. Cases of SF RDTs show the importance of postmarket surveillance of diagnostic kits. However, no studies trying to better understand the extent of quality issues of RDTs were identified.

Due to convenience, increasing accessibility to, perceived economical and confidential advantages of the internet, especially in the context of HIV/AIDS associated stigma and discriminations, online purchase of ARV is likely to increase. This may be particularly relevant to people searching for oral PrEP when at high risk of infection. In 2020, 130 countries had adopted the WHO recommendations on oral PrEP in national guidelines.^39^ Only two prevalence studies described the quality of ARV purchased on the internet, with too few samples collected to comment on the results.^40 41^

### Limitations

Searches were conducted only in English and French, risking the exclusion of articles, for example in Latin America, and we identified recalls/seizures/case reports mainly from searches in a limited number of MRA’s websites and other websites interested in medicine quality. Unpublished postmarketing surveillance results from other MRAs and the pharmaceutical industry were not captured. Most studies were of small sample size and used convenient sampling which risk bias. The quality of reporting of prevalence surveys was poor as reflected by the low MEDQUARG scores. The quality of samples was assessed by different pharmacopoeia references. In most prevalence surveys, we found limited information on stated country of manufacture and more than one-third of the samples were collected in one study in different outlets but no details on the quality of the samples by type of outlet were given. We, thus, did not perform further analysis that could lead to misleading interpretation.

The diversity of and the often poor methodology and reporting of the studies renders the findings of systematic reviews of medicine quality difficult to interpret and extrapolate,^30 31^ though we believe it is the best method to summarise the current evidence on the quality of different classes of medicines.

### Recommendations

There are clear gaps in the understanding of the epidemiology of SF ARV and related diagnostic tests. Initiatives such as the Distributed Pharmaceutical Analysis Laboratory (DPAL), a collaboration established between 30 academic institutions around the world to determine the quality of medicines collected from partner organisations in L/MICs, may facilitate better understanding of the epidemiology of SF medicines and other medical products.^42^ Although packaging analysis is difficult, especially in obtaining voucher samples, it is vital to allow the objective distinction between substandard and falsified products. That 92.2% of failing samples were classified as SorF is a major impediment for deciding on policy as interventions to counter substandard and falsified differ.

Key current global public health aims are the 95-95-95 target of the Sustainable Development Goals by 2026 and to end AIDS by 2030.^36 43^ Diagnosing 95% and achieving viral suppression in 95% of all HIV-positive individuals risks failure even if only 1% of the ARV/RDT available on the market do not fulfil their roles because they are poor quality. With millions of people being treated or using ARV for the prevention of HIV, even a small proportion of poor quality ARV with impaired efficacy or increased toxicity will greatly endanger the lives of millions, not only those treated, but also those who may be infected as a result of transmission from people using SF ARV. A related issue is concern about the quality of condoms, with many incidents and seizures of tons of falsified condoms with holes,^44–47^ but the extent of the problem is also unknown. Gaps in the scientific evidence impede development of objective action plans on how best to secure the supply chains for ARV, RDT and other medical devices such as condoms. With the current goals set by international actors to scale up community based approaches for both treatment and prevention, such as community drug distribution, safeguards to ensure quality ARV and RDT will be crucial. More efforts also need to be put into controlling the quality of medicines available on the internet.

Shortages of good quality ARV create opportunities for substandard and falsified ARV medicines to reach supply chains. Shortages are exacerbated during the COVID-19 pandemic, as land, sea and air transport services shut down. People had difficulties to access ARV because of travel restrictions, disruptions in health services within countries and worsening of the economic situation because of the pandemic.^48^ Better preparedness is needed for the next pandemic, for medical products to treat the pandemic’s causing agent and for other medical products vital to millions such as ARV.

In view of the limitations described above, prevalence surveys with robust survey methodology adequate sample sizes, and better reporting of findings, in wider geographical regions including HIC and online sales are needed for a more comprehensive epidemiological information on the quality of ARV medicines. This would allow examination of trends over time and the impact of SF ARV on humans and their economy.

## Conclusion

Even a small proportion of SF ARV is unacceptable, as it may result in a myriad of HIV positive people not receiving the correct treatment, risking poor outcomes and resistance, and those using ARV as prophylaxis unknowingly being unprotected against infection. These results cannot represent an exact prevalence of poor quality ARV drugs globally but are a warning sign. The methodological limitations do not allow exptrapolation that 1.4% of ARV globally are SF. There is clearly a risk and more data on the epidemiology of SF ARV, facilitation of packaging analysis and optimisation of devices for their screening of SF products in supply chains are needed.

## Data Availability

All data relevant to the study are included in the article or uploaded as online supplemental information. All data are mapped and can be downloaded on the Infectious Diseases Data Observatory Medicine Quality Surveyor system(https://www.iddo.org/mqsurveyor/%23antiretrovirals).

## References

[1] Saag MS , Benson CA , Gandhi RT , et al . Antiretroviral drugs for treatment and prevention of HIV infection in adults: 2018 recommendations of the International antiviral society-USA panel. JAMA 2018;320:379–96. 10.1001/jama.2018.8431 30043070 PMC6415748

[2] World Health Organization . HIV. Available: https://www.who.int/news-room/fact-sheets/detail/hiv-aids [Accessed 6 Sep 2022].

[3] UNICEF . HIV statistics - global and regional trends - UNICEF DATA 2022. Available: https://data.unicef.org/topic/hivaids/global-regional-trends/ [Accessed 15 Aug 2022].

[4] UNAIDS . Global HIV & AIDS statistics — 2020 fact sheet. Available: https://www.unaids.org/en/resources/fact-sheet [Accessed 7 Jul 2020].

[5] World Health Organization . HIV/AIDS. 2021. Available: https://www.who.int/news-room/fact-sheets/detail/hiv-aids [Accessed 7 Jul 2021].

[6] World Health Organization . Fact sheet: HIV drug resistance. Available: https://www.who.int/news-room/fact-sheets/detail/hiv-drug-resistance [Accessed 15 Sep 2022].

[7] World Health Organization . HIV drug resistance report 2021. Available: https://www.who.int/publications/i/item/9789240038608 [Accessed 15 Sep 2022].

[8] HIV Drugs Market . Global industry trends, share, size, growth, opportunity and forecast 2019-2024. Available: https://www.researchandmarkets.com/reports/4763052/hiv-drugs-market-global-industry-trends-share#rela4-4987308 [Accessed 7 Jul 2020].

[9] World Health Organization . WHO global surveillance and monitoring system for substandard and falsified medical products. 2017.

[10] Newton PN , Bond KC , Oxford Statement signatories . Global access to quality-assured medical products: the oxford statement and call to action. Lancet Glob Health 2019;7:e1609–11. 10.1016/S2214-109X(19)30426-7 31708137

[11] World Health Organization . A study on the public health and socioeconomic impact of substandard and falsified medical products. WHO 2018:4–8.

[12] Tabernero P , Fernández FM , Green M , et al . Mind the gaps -- the epidemiology of poor-quality anti-malarials in the malarious world -- analysis of the worldwide antimalarial resistance network database. Malar J 2014;13:139. 10.1186/1475-2875-13-139 24712972 PMC4021408

[13] Zabala GA , Bellingham K , Vidhamaly V , et al . Substandard and falsified antibiotics: neglected drivers of antimicrobial resistance? BMJ Glob Health 2022;7:e008587. 10.1136/bmjgh-2022-008587 PMC9394205 35981806

[14] Saraswati K , Sichanh C , Newton PN , et al . Quality of medical products for diabetes management: a systematic review. BMJ Glob Health 2019;4:e001636. 10.1136/bmjgh-2019-001636 PMC6768360 31637025

[15] Do NT , Bellingham K , Newton PN , et al . The quality of medical products for cardiovascular diseases: a gap in global cardiac care. BMJ Glob Health 2021;6:e006523. 10.1136/bmjgh-2021-006523 PMC8442059 34521627

[16] Machado Reis AT , Berardo BFR , Loureiro R . Quality of medicines in Portugal: a retrospective review of medicine recalls (2000-2015). PDA J Pharm Sci Technol 2018;72:44–9. 10.5731/pdajpst.2017.007567 29030530

[17] Naughton BD , Akgul E . Medicine quality in high-income countries: the obstacles to comparative prevalence studies. Med Access Point Care 2021;5:23992026211052270. 10.1177/23992026211052272 PMC9413605 36204504

[18] Almuzaini T , Sammons H , Choonara I . Quality of medicines in Canada: a retrospective review of risk communication documents (2005-2013). BMJ Open 2014;4:e006088. 10.1136/bmjopen-2014-006088 PMC4216865 25361839

[19] Almuzaini T , Sammons H , Choonara I . Substandard and falsified medicines in the UK: a retrospective review of drug alerts (2001-2011). BMJ Open 2013;3:e002924. 10.1136/bmjopen-2013-002924 PMC3731779 23883882

[20] AlQuadeib BT , Alfagih IM , Alnahdi AH , et al . Medicine recalls in Saudi Arabia: a retrospective review of drug alerts (January 2010–january 2019). Futur J Pharm Sci 2020;6:91. 10.1186/s43094-020-00112-3

[21] Penzak SR , Acosta EP , Turner M , et al . Antiretroviral drug content in products from developing countries. Clin Infect Dis 2004;38:1317–9. 10.1086/383575 15127347

[22] World Health Organization . Medicines regulatory authorities: current status and the way forward. 2006.

[23] CounterfeitDrugs Check combivir, serostim; Epogen

[24] Ravinetto R . [E-drug] counterfeit arvs in DRC. 2004. Available: http://lists.healthnet.org/archive/html/e-drug/2004-02/msg00028.html [Accessed 8 Jul 2020].

[25] World Health Organization . Member state mechanism on substandard/spurious/falsely-labelled/falsified/ counterfeit medical products. 2017.

[26] Vidhamaly V , Bellingham K , Newton PN , et al . The quality of veterinary medicines and their implications for one health. BMJ Glob Health 2022;7:e008564. 10.1136/bmjgh-2022-008564 PMC9351321 35918072

[27] Newton PN , Lee SJ , Goodman C , et al . Guidelines for field surveys of the quality of medicines: a proposal. PLoS Med 2009;6:e52. 10.1371/journal.pmed.1000052 19320538 PMC2659710

[28] India Kanoon . Calcutta high court (appellete side) - unknown vs the state of west bengal on 2014; 2014 Nov 14.

[29] BBC News . Kenya recalls ‘faulty’ south korean HIV kits. 2011. n.d. Available: https://www.bbc.com/news/world-africa-16355462?print=true

[30] McManus D , Naughton BD . A systematic review of substandard, falsified, unlicensed and unregistered medicine sampling studies: a focus on context, prevalence, and quality. BMJ Glob Health 2020;5:e002393. 10.1136/bmjgh-2020-002393 PMC7454198 32859648

[31] Mackey TK . Prevalence of substandard and falsified essential medicines: still an incomplete picture. JAMA Netw Open 2018;1:e181685. 10.1001/jamanetworkopen.2018.1685 30646099

[32] Mziray S , Maganda BA , Mwamwitwa K . Quality of selected anti-retroviral medicines: tanzania mainland market as a case study. Springer, Available: link.springer.com/article/10.1186/s40360-021-00514-w 10.1186/s40360-021-00514-w PMC8390223 34446094

[33] Vickers S , Bernier M , Zambrzycki S , et al . Field detection devices for screening the quality of medicines: a systematic review. BMJ Glob Health 2018;3:e000725. 10.1136/bmjgh-2018-000725 PMC6135480 30233826

[34] Cohn J , von Schoen-Angerer T , Jambert E , et al . When falsified medicines enter the supply chain: description of an incident in Kenya and lessons learned for rapid response. J Public Health Policy 2013;34:22–30. 10.1057/jphp.2012.53 23172047

[35] Amon JJ . Dangerous medicines: unproven AIDS cures and counterfeit antiretroviral drugs. Global Health 2008;4:5. 10.1186/1744-8603-4-5 18304316 PMC2291042

[36] UNAIDS . Fast-track ending the AIDS epidemic by 2030. Available: https://www.unaids.org/sites/default/files/media_asset/JC2686_WAD2014report_en.pdf [Accessed 7 Nov 2022].

[37] World Health Organization . Global HIV programme - treatment & care. Available: https://www.who.int/teams/global-hiv-hepatitis-and-stis-programmes/hiv/treatment [Accessed 7 Nov 2022].

[38] World Health Organization . Global HIV programme - pre-exposure prophylaxis. Available: https://www.who.int/teams/global-hiv-hepatitis-and-stis-programmes/hiv/prevention/pre-exposure-prophylaxis [Accessed 7 Nov 2022].

[39] World Health Organization . Global state of prep. Available: https://www.who.int/groups/global-prep-network/global-state-of-prep [Accessed 7 Nov 2022].

[40] Wang T , Hoag SW , Eng ML , et al . Quality of antiretroviral and opportunistic infection medications dispensed from developing countries and Internet pharmacies. J Clin Pharm Ther 2015;40:68–75. 10.1111/jcpt.12226 25381836

[41] Wang X , Nutland W , Brady M , et al . Quantification of tenofovir disoproxil fumarate and emtricitabine in generic pre-exposure prophylaxis tablets obtained from the Internet. Int J STD AIDS 2019;30:765–8. 10.1177/0956462419841144 31072205

[42] Bliese SL , Berta M , Lieberman M . Involving students in the distributed pharmaceutical analysis laboratory: a citizen-science project to evaluate global medicine quality. J Chem Educ 2020;97:3976–83. 10.1021/acs.jchemed.0c00904 33840832 PMC8026146

[43] UNAIDS . Global AIDS strategy 2021-2026 — end inequalities. end AIDS. Available: www.unaids.org/en/resources/documents/2021/2021-2026-global-AIDS-strategy [Accessed 8 Nov 2022].

[44] The New Humanitarian . Vietnam’s counterfeit condom crisis. Available: https://www.thenewhumanitarian.org/analysis/2014/06/09/vietnam-s-counterfeit-condom-crisis [Accessed 8 Nov 2022].

[45] Daily Mail Online . Fake condoms warning. Available: https://www.dailymail.co.uk/health/article-341766/Fake-condoms-warning.html [Accessed 8 Nov 2022].

[46] SecuringIndustry.com . Too short’ condoms lead to counterfeit bust in spain. Available: https://www.securingindustry.com/cosmetics-and-personal-care/-too-short-condoms-lead-to-counterfeit-bust-in-spain/s106/a11930/#.Y2povHbP1hE [Accessed 8 Nov 2022].

[47] Counterfeit condom gang in china broken up. Available: https://www.businessinsider.com/counterfeit-condom-gang-in-china-broken-up-2018-11?r=US&IR=T [Accessed 8 Nov 2022].

[48] World Health Organization . WHO: access to HIV medicines severely impacted by COVID-19 as AIDS response stalls 2020. Available: https://www.who.int/news/item/06-07-2020-who-access-to-hiv-medicines-severely-impacted-by-covid-19-as-aids-response-stalls [Accessed 8 Nov 2022].

