# Supplementary material for: The quality of antiretroviral medicines: an uncertain problem
Source: BMJ Glob Health. 2023 Mar 15;8(3):e011423. doi: 10.1136/bmjgh-2022-011423 (PMC10030546; doi:10.1136/bmjgh-2022-011423)
Supplement: Supplementary data [file bmjgh-2022-011423supp001.pdf]

Supplementary file 1: Search terms used for each source in the systematic review of the quality of ARV medicines

Sources	English search Terms
Pubmed	(“quality control” OR “drug quality” OR “quality analysis” OR counterfeit OR fake OR falsified OR spurious OR substandard OR “medicine quality” OR “pharmaceutical quality”) AND (antiretroviral OR anti-retroviral OR ARV OR HIV OR “Human Immunodeficiency Virus” OR AIDS OR “Acquired Immune Deficiency Syndrome”)
Embase	('quality control' OR 'drug quality' OR 'quality analysis' OR counterfeit OR fake OR falsified OR spurious OR substandard OR 'medicine quality' OR 'pharmaceutical quality') AND (antiretroviral OR anti-retroviral OR ARV OR HIV OR 'Human Immunodeficiency Virus' OR AIDS OR 'Acquired Immune Deficiency Syndrome')
Web of Science	('quality control' OR 'drug quality' OR 'quality analysis' OR counterfeit OR fake OR falsified OR spurious OR substandard OR 'medicine quality' OR 'pharmaceutical quality') AND (antiretroviral OR anti-retroviral OR ARV OR HIV OR 'Human Immunodeficiency Virus' OR AIDS OR 'Acquired Immune Deficiency Syndrome')
Google Scholar	(substandard OR “medicine quality” OR “pharmaceutical quality”) AND (anti-retroviral OR AIDS)
	(“quality control” OR “drug quality ” OR “quality analysis”) AND (“Human Immunodeficiency Virus”)
	(counterfeit OR fake OR falsified OR spurious) AND (antiretroviral OR anti-retroviral OR ARV OR HIV)
	(substandard OR “medicine quality” OR “pharmaceutical quality”) AND (“Human Immunodeficiency Virus”)
	(“quality control” OR “drug quality” OR “quality analysis”) AND (antiretroviral OR anti-retroviral)
	(“quality control” OR “drug quality” OR “quality analysis”) AND (ARV OR HIV OR AIDS)
	(counterfeit OR fake OR falsified OR spurious) AND (“Human Immunodeficiency Virus” OR AIDS)
	(counterfeit OR fake OR falsified OR spurious) AND (“Acquired Immune Deficiency Syndrome”)
	(substandard OR “medicine quality” OR “pharmaceutical quality”) AND (antiretroviral OR ARV OR HIV)
	(“pharmaceutical quality”) AND (“Acquired Immune Deficiency Syndrome”)
	(substandard OR “medicine quality”) AND (“Acquired Immune Deficiency Syndrome”)
Google	(“quality control” OR “drug quality” OR “quality analysis” OR counterfeit OR fake OR falsified OR spurious OR substandard OR “medicine quality” OR “pharmaceutical quality”) AND (antiretroviral OR anti-retroviral OR ARV OR HIV OR “Human Immunodeficiency Virus” OR AIDS OR “Acquired Immune Deficiency Syndrome”)
**Sources**	**French search Terms**
	(“contrôle qualité” OR "qualité des médicaments" OR “analyse de qualité”) AND (antirétroviral)
	(sous-standard OR “ qualité de médicament” OR “qualité pharmaceutique”) AND (antirétroviral)
	("faussement étiqueté" OR contrefait) AND (“syndrome d'immunodéficience acquise”)
	("faussement étiqueté" OR contrefait) AND ("virus de l'immunodéficience humaine")

Google Scholar	(“contrôle qualité” OR "qualité des médicaments" OR “analyse de qualité”) AND (antirétroviral)
	(“contrôle qualité” OR "qualité des médicaments") AND (“syndrome d'immunodéficience humaine)
	(sous-standard) AND ("virus de l'immunodéficience humaine" OR “syndrome d'immunodéficience acquise")
	(contrefaçon OR "faux médicament" OR "médicament fallacieux" OR falsifié) AND (ARV OR VIH OR SIDA)
	(sous-standard OR “ qualité de médicament” OR “qualité pharmaceutique”) AND (antirétroviral)
	(“qualité de médicament” OR “qualité pharmaceutique”) AND ("virus de l'immunodéficience humaine)
	("faux médicament" OR "médicament fallacieux") AND (“syndrome d'immunodéficience acquise”)
	(“contrôle qualité” OR "qualité des médicaments") AND ("virus de l'immunodéficience humaine")
	("faussement étiqueté" OR contrefait) AND (antirétroviral)
	(“qualité de médicament” OR “qualité pharmaceutique”) AND (“syndrome d'immunodéficience acquise”)
	(“analyse de qualité” OR contrefaçon OR falsifié) AND ("virus de l'immunodéficience humaine")
	("faux médicament" OR "médicament fallacieux") AND ("virus de l'immunodéficience humaine)
	("faussement étiqueté" OR contrefait) AND (ARV OR VIH OR SIDA)
	(“analyse de qualité” OR contrefaçon OR falsifié) AND (“syndrome d'immunodéficience acquise”)
	(“contrôle qualité” OR "qualité des médicaments" OR “analyse de qualité”) AND (ARV OR VIH OR SIDA)
	(contrefaçon OR "faux médicament" OR "médicament fallacieux" OR falsifié) AND (antirétroviral)
	(sous-standard OR “qualité de médicament” OR “qualité pharmaceutique”) AND (ARV OR VIH OR SIDA)
Google	(“contrôle qualité” OR "qualité des médicaments" OR “analyse de qualité” OR contrefaçon OR "faux médicament" OR "médicament fallacieux" OR falsifié OR sous-standard OR “qualité de médicament” OR “qualité pharmaceutique” OR "faussement étiqueté" OR contrefait) AND (antirétroviral OR antirétroviral OR ARV OR VIH)
	(“contrôle qualité” OR "qualité des médicaments" OR “analyse de qualité” OR contrefaçon OR "faux médicament" OR "médicament fallacieux" OR falsifié OR sous-standard OR “qualité de médicament” OR “qualité pharmaceutique” OR "faussement étiqueté" OR contrefait) AND ("virus de l'immunodéficience humaine" OR SIDA)
PubMed	(“contrôle qualité” OR "qualité des médicaments" OR “analyse de qualité” OR contrefaçon OR "faux médicament" OR "médicament fallacieux" OR falsifié OR sous-standard OR “qualité de médicament” OR “qualité pharmaceutique” OR "faussement étiqueté" OR contrefait) AND (antirétroviral OR antirétroviral OR ARV OR VIH OR "virus de l'immunodéficience humaine" OR “syndrome d'immunodéficience acquise ”OR SIDA)
Embase	(“contrôle qualité” OR "qualité des médicaments" OR “analyse de qualité” OR contrefaçon OR "faux médicament" OR "médicament fallacieux" OR falsifié OR sous-standard OR “qualité de médicament” OR “qualité pharmaceutique” OR "faussement étiqueté" OR contrefait) AND

	(antirétroviral OR antirétroviral OR ARV OR VIH OR "virus de l'immunodéficience humaine" OR “syndrome d'immunodéficience acquise ”OR SIDA)
Web of Science	(“contrôle qualité” OR "qualité des médicaments" OR “analyse de qualité” OR contrefaçon OR "faux médicament" OR "médicament fallacieux" OR falsifié OR sous-standard OR “qualité de médicament” OR “qualité pharmaceutique” OR "faussement étiqueté" OR contrefait) AND (antirétroviral OR antirétroviral OR ARV OR VIH OR "virus de l'immunodéficience humaine" OR “syndrome d'immunodéficience acquise ”OR SIDA)
