# Supplementary material for: The quality of antiretroviral medicines: an uncertain problem
Source: BMJ Glob Health. 2023 Mar 15;8(3):e011423. doi: 10.1136/bmjgh-2022-011423 (PMC10030546; doi:10.1136/bmjgh-2022-011423)
Supplement: Supplementary data [file bmjgh-2022-011423supp002.pdf]

Supplementary file 2. Websites used for information gathering about substandard and falsified antiretroviral medicines

	**Websites names and hyperlinks**		**Websites names and hyperlinks**
**International Organisations and NGOs**	++ Health Action International ++	**Medicine Regulatory Authorities and national bodies**	Centers for Disease Control and Prevention
	ACG - Anti-counterfeting group		Comité national anti-contrefaçon
	Coalition Against Illicit Trade		Fraud.org
	EAASM-European Alliance for Access to Safe Medicines		NAFDAC Nigeria
	Fondation Chirac - Agir au service de la paix		Ghana FDA
	GACG Global Anti-Counterfeiting Network		HSA Health Sciences Authority Singapore
	GPHF The Global Pharma Health Fund		Medicines and Healthcare products Regulatory Agency, UK Government
	IACC-International AntiCounterfeiting Coalition		US Food and Drug Administration
	Medical Products Counterfeiting and Pharmaceutical Crime (INTERPOL)		Kenya Pharmacy and poisons Board
	IRACM Institut de Recherche Anti-Contrefaçon de Médicaments		Central Drugs Standard Control Organization
	L'Office des Nations unies contre la drogue et le crime		Ordre national des pharmaciens de côte d'ivoire
	MIMS		
	MSF Access Campaign msfaccess.org	**Alert lists and systems**	Campaign for Safe Medicines in Kenya
	Permanent Forum on International Pharmaceutical Crime		Minilabs save lives
	PhaReD Foundation - Home		mPedigree Network Bringing Quality To Life Innovative Global Partnerships Against Fake Drugs
	Pharmelp Detection of counterfeit medicines		Pharmabiz
	ReMeD-Réseau Médicaments & Développement		PharmaSecure
	Safe Medicines India		Sproxil Protecting Brands Globally
	Safemedicines Protecting the Safety of America's Drug Supply		Association Développement et Santé
	The Medicrime Convention		ContrefaçonRiposte
	Third World Network (TWN)		
	USP Promoting the Quality of Medicines (PQM)		
**Academic/ Research Initiatives**	QUAMED - Quality Medicines for All	**Newspaper websites with interest in**	allafrica.com
	IRASEC- Institut de Recherche sur l'Asie du Sud-Est Contemporaine		Daily Monitor - Uganda News, Politics, Business, Travel, Health, Sports, News Paper, technology
			Ghanaweb
			L'ESSOR journal Mali

**Pharmaceutical Industry**	Sanofi	**medicine quality**	ModernGhana
	Les entreprises du médicament		Nigerian Tribune
	Pfizer Pharmaceutical News and Media Pfizer One of the world's premier biopharmaceutical companies		Rapideinfo-Journal Mauritanie
	PSI-Inc.org		THE HANS INDIA
