# Supplementary material for: The quality of antiretroviral medicines: an uncertain problem
Source: BMJ Glob Health. 2023 Mar 15;8(3):e011423. doi: 10.1136/bmjgh-2022-011423 (PMC10030546; doi:10.1136/bmjgh-2022-011423)
Supplement: Supplementary data [file bmjgh-2022-011423supp003.pdf]

**Supplementary file 3: Types of studies included in the review and definition**		
	**Study/report type**	**Definition**
**Scientific reports**	Quality control	Study in which samples were collected to be analyzed in routine post-marketing surveillance by MRAs or a laboratory mandated by MRAs
	Prevalence survey	Study in which samples were collected within the pharmaceutical supply chain to assess their quality, to describe the prevalence of circulating SF medicines
	Equivalence study	Study to assess the quality of different marketed brands of the same API(s) assuming that the results of the collected samples would represent the quality of the brand as a whole and not an estimate of the frequency of individual samples of different quality
	Analysis technique development/validation	Study in which samples are assembled in a laboratory to answer a chemical, rather than an epidemiological question (mostly for the development of a new quality technique)
	Bioavailability study	Study of the in vivo bioavailability, i.e. testing for adequate body tissue concentration including the rate and extent to which drug reaches the body tissue compartment
**Other reports**	Recall/warning/alert	Recall/Warning/Alert of products by manufacturers via MRA or by MRAs directly, or by WHO rapid alert
	Case reports	Patients not responding to medicines or adverse drug reactions where the quality of the medicine was suspected as the cause. Also includes samples analyzed for quality not included in a scientific study.
	Seizure	Confiscations by police or MRA
**API, Active Pharmaceutical Ingredient; MRA, Medicines Regulatory Agency; WHO, World Health Organization**		
