# Supplementary material for: The quality of antiretroviral medicines: an uncertain problem
Source: BMJ Glob Health. 2023 Mar 15;8(3):e011423. doi: 10.1136/bmjgh-2022-011423 (PMC10030546; doi:10.1136/bmjgh-2022-011423)
Supplement: Supplementary data [file bmjgh-2022-011423supp005.pdf]

Supplementary file 5 : Number of publications, data points and samples per study/report type in publications (either scientific studies or other reports) containing description of the quality of antiretroviral medicines/medical devices in data point(s), i.e. in a specific location at a specific time

	**Study type**	**No. publications n (%)**	**No. data points n (%)**	**No. samples n (%)**
**Scientific reports**	Prevalence survey	19 (25.0%)	168 (36.8%)	3713 (75.8%)
	Quality control	8 (10.5%)	46 (10.1%)	766 (15.6%)
	Analytical technique development/validation	15 (19.7%)	20 (4.4%)	35 (0.7%)
	Equivalence study	4 (5.3%)	5 (1.1%)	18 (0.4%)
	Bioavailability studies	1 (1.3%)	2 (0.4%)	0 (0.0%)
	Unknown*	1 (1.3%)	177 (38.8%)	366 (7.5%)
	**Total**	**48 (63.2%)**	**418 (91.7%)**	**4,898 (100%)**
**Other reports**	Recall/warning/alert	16 (21.1%)	21 (4.6%)	
	Seizure	7 (9.2%)	10 (2.2%)	
	Case reports	5 (6.6%)	7 (1.5%)	
	**Total**	**28 (36.8%)**	**36 (8.3%)**	
**Total**		**76 (100.0%)**	**456 (100.0%)**	

*Data from the Medicines Quality Database (MQDB) of United States Pharmacopeia
