# Supplementary material for: The quality of antiretroviral medicines: an uncertain problem
Source: BMJ Glob Health. 2023 Mar 15;8(3):e011423. doi: 10.1136/bmjgh-2022-011423 (PMC10030546; doi:10.1136/bmjgh-2022-011423)
Supplement: Supplementary data [file bmjgh-2022-011423supp006.pdf]

Supplementary file 6: Main Characteristics of prevalence surveys of antiretroviral medicines

Reference / country of collection	Active pharmaceutical ingredient (API)	Brand name	Outlet/ Sampling type	Reference specifications stassindard	Test (s) performed and main analytical technique	No. Failed n (%)
Penzak, S.R. 2003 [1] Kenya, Lithuania, Zambia, South Africa	Nevirapine	Triomune 40<br>Nevimune(200)<br>Nevirex<br>Viramune (Nev 200)<br>Triomune 30	Private pharmacy/ Convenience	Unspecified	API content<br><br>*High-performance liquid chromatography /ultraviolet (HPLC-UV)*	0 /6 (0.0%)
Ramachandran, G. 2004 [2] Zimbabwe, South Africa, Lithuania, Jamaica, Zambia	Efavirenz<br>Indinavir<br>Lopinavir-ritonavir<br>Amprenavir<br>Ritonavir<br>Saquinavir soft-gel capsules	Sustiva<br>Stocrin<br>Crixivan<br>Indivex-400<br>Kaletra<br>Agenerase<br>Norvir<br>Fortovase	Unspecified/ Convenience	United States Pharmacopeia (USP) /National Formulary	API content<br><br>*HPLC-UV*	3/12 (25.0%)
Ramachandran, G, 2004 [3] India	Lamivudine-Nevirapine-Stavudine<br>Efavirenz<br>Nevirapine<br>Zidovudine<br>Stavudine<br>Lamivudine<br>Didanosin	Unspecified	Manufacturer/ Convenience	Unspecified	API content<br><br>*HPLC-UV*	0/17 (0.0%)
Ciss, M. 2006 [4] Senegal	Stavudine<br>Nevirapine<br>Indinavir	Unspecified	Combination of outlets / Convenience	USP + European Pharmacopeia + International Pharmacopoeia	API content<br><br>*HPLC-unknown detector*	1/12 (8.3%)
M.H.Chambuso, 2006 [5] Tanzania	Indinavir<br>Stavudine	Unspecified	Private pharmacy/ Unspecified	British Pharmacopoeia (BP) + USP	API content, API identification, Dissolution, Visual inspection<br><br>*UV-visible spectrophotometry*	4/5 (80.0%)
WHO, 2007 [6] Uganda, Tanzania,	Didanosin, Efavirenz, Lamivudine, Nevirapine, Stavudine, Zidovudine,	Unspecified	Combination of outlets / Random	Brazilian Pharmacopeia, USP 2008	API content, Content uniformity, Dissolution, weight, Visual inspection, impurity, API identification	8/394 (2.0%)

**Reference / country of collection**	**Active pharmaceutical ingredient (API)**	**Brand name**	**Outlet/ Sampling type**	**Reference specifications stassindard**	**Test (s) performed and main analytical technique**	**No. Failed n (%)**
Democratic Republic of the Congo, Zambia, Kenya, Nigeria, Cameroon	Lamivudine-Zidovudine, Lamivudine-Nevirapine-Stavudine				*HPLC-UV/Vis*	
Sarr, S. O. 2008 [7] Senegal	Didanosin<br>Indinavir<br>Lamivudine<br>Nevirapine<br>Stavudine<br>Zidovudine	Unspecified	Hospital/health centres / Convenience	Unspecified	API content (semi-quantitation), Disintegration, Visual inspection<br><br>*Thin Layer Chromatography (TLC)*	7/43 (16.3%)
Musoke. D 2008 [8]<br>Uganda	Lamivudine-Nevirapine-Stavudine	Unspecified	Hospital/health centres / Random	USP	API content, Visual inspection, dissolution<br><br>*HPLC-Ultra-Violet*	0/14 (0.0%)
Munkombwe, D 2010 [9] Zambia	Nevirapine<br>Lamivudine-Nevirapine-Stavudine<br>Efavirenz<br>Lamivudine-Stavudine	Unspecified	Government outlets/ Random	BP + USP	API content, API identification<br><br>*TLC*	1/11 (9.1%)
NASCOP 2012 [10] Kenya	Abacavir<br>Abacavir-Lamivudine<br>Didanosin<br>Efavirenz<br>Emtricitabine-Efavirenz-Tenofovir Disiproxil<br>Lamivudine<br>Lamivudine-Stavudine<br>Lamivudine-Nevirapine-Stavudine<br>Lamivudine-Zidovudine<br>Lamivudine-Zidovudine-Nevirapine<br>Nevirapine<br>Stavudine<br>Tenofovir Disoproxil<br>Tenofovir disoproxil – Lamivudine	Unspecified	Combination of outlets / Convenience	BP + USP	API content, Dissolution, weight, Friability<br><br>*HPLC- Unknown detector*	1/284 (0.4%)

Reference / country of collection	Active pharmaceutical ingredient (API)	Brand name	Outlet/ Sampling type	Reference specifications stassindard	Test (s) performed and main analytical technique	No. Failed n (%)
	Zidovudine<br>Lopinavir-Ritonavir					
Chigunta, M.[11] 2013 Zambia	Nevirapine	Unspecified	Combination of outlets/ Convenience	USP	API content , API identification, Visual inspection<br><br>*HPLC – unknown detector*	0/9 (0.0%)
Krech. L.A. 2014 [12] Cambodia	Lamivudine-Nevirapine-Stavudine	Unspecified	Combination of outlets/ Convenience	USP and Cambodia Pharmacopeia	Visual inspection, API content , API identification, Impurity, Disintergration<br><br>*HPLC, TLC*	1/7 (14.3%)
Wang, T. 2015 [13]<br>Thailand, Nigeria, Ethiopia, China, United States of America, South Africa	Lamivudine<br>Nevirapine<br>Zidovudine<br>Efavirenz	Unspecified	Government outlets, Non-governmental organization, Website, Private pharmacy / Convenience	USP	API content, Visual inspection, Impurity<br><br>*HPLC-UV*	6/46 (13.0%)
Sapsirisavat, V. 2016 [14]<br>Thailand, Viet Nam	Lopinavir-Ritonavir<br>Tenofovir<br>Efavirenz		Combination of outlets / Random and convenience	WHO International Pharmacopoeia	API content, API identification, Uniform of mass, Dissolution<br><br>*Pharmacopeial techniques followed, no detail of the specific techniques*	0/42 (0.0%)
Kamangu, E.N. 2017 [15]<br>Democratic Republic of the Congo	Abacavir<br>Zidovudine<br>Efavirenz<br>Nevirapine	Nevipan (200)<br>Nevimune (200)<br>Nevirapine (200)<br>EstivaN<br>Aviroz<br>Abacavir sulfate	Hospital/health centres / Convenience	European Pharmacopoeia, BP, International Pharmacopoeia 6th Edition.	API content, Disintegration, Friability<br><br>*UV-visible spectrophotometry*	1/10 (10.0%)
WHO 2017 [16]<br>Burkina Faso, Democratic Republic of the	Unspecified	Unspecified	Combination of outlets / Convenience	BP+USP+ International Pharmacopoeia	Where applicable: Appearance, API identification, API content , Weight variation, Dissolution, Impurity, Related substances, Fineness of	1/126 (0.8%)

**Reference / country of collection**	**Active pharmaceutical ingredient (API)**	**Brand name**	**Outlet/ Sampling type**	**Reference specifications stassindard**	**Test (s) performed and main analytical technique**	**No. Failed n (%)**
Congo (DRC), Nigeria, Rwanda and Zambia.					dispersion, Water content – no details on results per test<br><br>*TLC, HPLC -unknown detector, IR (depending on API and formulation)*	
Djobet. M.P.N 2017 [17] Cameroon	Efavirenz<br>Lamivudine-Stavudine<br>Nevirapine<br>Lamivudine-Zidovudine-Nevirapine<br>Lamivudine-Zidovudine	Unspecified	Combination of outlets / Convenience	USP	API content , API identification, Visual inspection, Disintegration<br><br>*UV Spectrophotometry*<br>*TLC*	0/35 (0.0%)
Wang, X 2019 [18] United Kingdom	Emtricitabine-Tenofovir Disoproxil	Unspecified	Website , Hospital/health centers / Convenience	Unspecified	API content , API identification<br><br>*Ultra-Performance Liquid Chromatography (UPLC)*	0/16 (0.0%)
Mziray S 2021 [19] Tanzania	Efavirenz<br>Nevirapine<br>Lamivudine<br>Zidovudine<br>Tenofovir Disoproxil Fumarate<br>Lamivudine/Zidovudine<br>Abacavir Sulphate<br>Lopinavir/Ritonavir<br>Tenofovir Disoproxil Fumarate /Emtricitabin<br>Tenofovir Disoproxil Fumarate /Lamivudine/ Efavirenz<br>Lamivudine/Zidovune/Nevirapine<br>Lamivudine/Stavudine/ Nevirapine<br>Tenofovir Disoproxil Fumarate /Efavirenz/ Emtricitabine	Unspecified	Combination of outlets / Convenience	USP or manufacturer's methods or in-house specifications	API content, API identification, Disintegration, Dissolution, Visual inspection, Related substances, Weight uniformity<br><br>*TLC and HPLC*<br><br>*Tier analysis:*<br>*Tier 1 : screening testing using the GPHF-Minilab*<br>*Tier 2 : samples failing Tier 1 and 10% samples not failing Tier 1 followed full pharmacopeial testing*	17/2630 (0.6%)

Reference / country of collection	Active pharmaceutical ingredient (API)	Brand name	Outlet/ Sampling type	Reference specifications stassindard	Test (s) performed and main analytical technique	No. Failed n (%)
HPLC, High-performance liquid chromatography; IR, Infrared; UV, Ultraviolet; TLC, Thin Layer Chromatography; BP, British Pharmacopeia; USP, United Stated Pharmacopeia; API, Active Pharmaceutical Ingredient						

1 Penzak SR, Acosta EP, Turner M, *et al.* Analysis of Generic Nevirapine Products in Developing Countries. *J Am Med Assoc* 2003;**289**:2648–9. doi:10.1001/jama.289.20.2648-c

2 Penzak SR, Acosta EP, Turner M, *et al.* Antiretroviral Drug Content in Products from Developing Countries. *Clin Infect Dis* 2004;**38**:1317–9. doi:10.1086/383575

3 Ramachandran G, Perloff ES, Von Moltke LL, *et al.* Analysis of generic antiretroviral formulations manufactured in India. AIDS. 2004;**18**:1482–4. doi:10.1097/01.aids.0000131346.76289.27

4 Ciss M. Une démarche qualité pour le contrôle des médicaments: Cas des ARV. 22/03/2006 Pr M. - PDF Free Download. https://docplayer.fr/21057770-Laboratoire-national-de-controle-des-medicaments-une-demarche-qualite-pour-le-controle-des-medicaments-cas-des-arv-22-03-2006-pr-m.html (accessed 2 Jun 2020).

5 Chambuso MH, Ngassapa OD, Sayi JG, *et al.* Quality of antiretroviral drugs, stavudine and indinavir capsules available in the Tanzanian market. *Tanzania Med J* 2006;**21**:8–12. doi:10.4314/TMJ.V21I1.39202

6 WHO. Survey of the quality of antiretroviral medicines circulating in selected African countries. https://extranet.who.int/prequal/content/ (accessed 2 Jun 2020).

7 Sarr SO. Control of the Quality of Antiretroviral Medicines Used in Senegal. 2008.https://pubmed.ncbi.nlm.nih.gov/19626794/ (accessed 2 Jun 2020).

8 David Musoke. Assessment of the drug storage facilities and quality of generic co-formulation tablets (stavudine, lamivudine, nevirapine) at HIV/AIDS treatment centers in Uganda. https://www.researchgate.net/publication/289843470_Assessment_of_the_drug_storage_facilities_and_quality_of_generic_co-

formulation_tablets_stavudine_lamivudine_nevirapine_at_HIVAIDS_treatment_centers_in_Uganda (accessed 2 Jun 2020).

9 Munkombwe D. Quality analysis of some first-line HIV/AIDS medicines dispensed in Lusaka District Health facilities of Zambia. Published Online First: 16 November 2011.http://palevel.unza.zm/handle/123456789/897 (accessed 2 Jun 2020).

10 NASCOP. Post Market Survey of Antiretroviral Medicines in Kenya. https://drive.google.com/file/d/1KlPAstp8su82rS_bTWfMfmRSRDL93QbM/view (accessed 2 Jun 2020).

11 Chigunta MM. Quality analysis of selected Pediatric HIV/AIDS/TB Medicines in Livingstone District, Zambia. Published Online First: 4 February 2014.http://dspace.unza.zm:8080/xmlui/bitstream/handle/123456789/3188/Chigunta.pdf?sequence=1 (accessed 2 Jun 2020).

12 Christi Lane LAK. Cambodian Ministry of Health Takes Decisive Actions in the Fight against Substandard and Counterfeit Medicines. *Trop Med Surg* 2014;**02**. doi:10.4172/2329-9088.1000166

13 Wang T, Hoag SW, Eng ML, *et al.* Quality of antiretroviral and opportunistic infection medications dispensed from developing countries and Internet pharmacies. *J Clin Pharm Ther* 2015;**40**:68–75. doi:10.1111/jcpt.12226

14 Sapsirisavat V, Vongsutilers V, Thammajaruk N, *et al.* Pharmaceutical Equivalence of Distributed Generic Antiretroviral (ARV) in Asian Settings: The Cross-Sectional Surveillance Study – PEDA Study. *PLoS One* 2016;**11**:e0157039. doi:10.1371/JOURNAL.PONE.0157039

15 Kamangu EN, Mbinze JK, Mingu AM, *et al.* UV Visible Spectrophotometric Determination of the Quality of Antiretroviral Drugs Distributed in Kinshasa. *OALib* 2017;**04**:1–8. doi:10.4236/oalib.1102923

16 World Health Organization. Survey of the quality of selected antiretroviral medicines circulating in five African countries. Published Online First: 2017.https://extranet.who.int/prequal/content/ (accessed 2 Jun 2020).

17 Djobet MPN, Singhe D, Lohoue J, *et al.* Antiretroviral therapy supply chain quality control and assurance in improving people living with HIV therapeutic

outcomes in Cameroon. *AIDS Res Ther* 2017;**14**:19. doi:10.1186/s12981-017-0147-x

18 Wang X, Nutland W, Brady M, *et al.* Quantification of tenofovir disoproxil fumarate and emtricitabine in generic pre-exposure prophylaxis tablets obtained from the internet. *Int J STD AIDS* 2019;**30**:765–8. doi:10.1177/0956462419841144

19 Mziray S, Maganda BA, and … KM-BMCP, *et al.* Quality of selected anti-retroviral medicines: Tanzania Mainland market as a case study. *Springer*https://link.springer.com/article/10.1186/s40360-021-00514-w
