# Supplementary material for: The quality of antiretroviral medicines: an uncertain problem
Source: BMJ Glob Health. 2023 Mar 15;8(3):e011423. doi: 10.1136/bmjgh-2022-011423 (PMC10030546; doi:10.1136/bmjgh-2022-011423)
Supplement: Supplementary data [file bmjgh-2022-011423supp007.pdf]

Supplementary file 7: Failure rate per type of quality test performed in prevalence surveys

Because of the limited number of samples tested for quality in the studies included in this review, the figures should not be interpreted as representative of the prevalence of specific SF antiretroviral medicines (please refer to the discussion section of the current paper for more details)

**Quality test**	**Failure Rate % (n/N)**
API content	1.4% (14/1,034)
Dissolution	1.3% (8/616)
Visual inspection of dosage units/non-comparative packaging analysis	0.2% (6/3,256)
Impurity/Contaminant/Related substance	0.0% (0/495)
Other physical analysis**	0.5% (19/3,910)
Other chemical analysis*	0.2% (5/2,830)
Unknown/Not detailed	0.8% (1/133)

**Includes content uniformity, bioavailability, identification of APIs, API semi-quantitation*

***Includes weight uniformity, weight variation, friability, hardness, disintegration, pH, microbiology, mass uniformity*

Note: One sample may have been tested for one or more quality tests
