# Supplementary material for: The quality of antiretroviral medicines: an uncertain problem
Source: BMJ Glob Health. 2023 Mar 15;8(3):e011423. doi: 10.1136/bmjgh-2022-011423 (PMC10030546; doi:10.1136/bmjgh-2022-011423)
Supplement: Supplementary data [file bmjgh-2022-011423supp008.pdf]

| **Supplementary file 8:** Samples collected by type of outlet in prevalence surveys<br>*Because of the limited number of samples tested for quality in the studies included in this review, the figures should not be interpreted as representative of the prevalence of specific SF antiretroviral medicines (please refer to the discussion section of the current paper for more details)* | |
|---|---|
| **Type of outlet** | **Failure rate % (n/N)** |
| Private pharmacy | 28.0% (7/25) |
| Hospital/health centres | 19% (8/98) |
| Website | 7.7% (2/26) |
| Government outlets – others* | 6.3% (1/16) |
| Combination of outlets | 2.2% (29/1,302) |
| Ports of entry | 0.0% (0/2,200) |
| Manufacturer | 0.0% (0/17) |
| Non-governmental organization | 0.0% (0/7) |
| Wholesalers/importer/distributors | 0.0% (0/1) |
| Unknown | 19.0% (4/21) |
| **Total** | **1.4% (51/3,713)** |
| *included: *government pharmacies and other public health facilities* | |
