# Supplementary material for: The quality of antiretroviral medicines: an uncertain problem
Source: BMJ Glob Health. 2023 Mar 15;8(3):e011423. doi: 10.1136/bmjgh-2022-011423 (PMC10030546; doi:10.1136/bmjgh-2022-011423)
Supplement: Supplementary data [file bmjgh-2022-011423supp009.pdf]

Supplementary file 9: Failure rates by region of the stated manufacturer in prevalence surveys

Because of the limited number of samples tested for quality in the studies included in this review, the figures should not be interpreted as representative of the prevalence of specific SF antiretroviral medicines (please refer to the discussion section of the current paper for more details)

**Continent**	**Failure Rate n/N (%)**
Americas	14.3% (1/7)
Asia	3.8% (9/238)
Europe	0.0% (0/2)
Africa	0.0% (0/2)
Unknown	1.2% (41/3,464)
**Total**	1.4% (51/3,713)
