# Supplementary material for: The quality of antiretroviral medicines: an uncertain problem
Source: BMJ Glob Health. 2023 Mar 15;8(3):e011423. doi: 10.1136/bmjgh-2022-011423 (PMC10030546; doi:10.1136/bmjgh-2022-011423)
Supplement: Supplementary data [file bmjgh-2022-011423supp010.pdf]

Supplementary file 10. Frequency of agreement with the 26 items of the MEDQUARG checklist of the 15 prevalence surveys

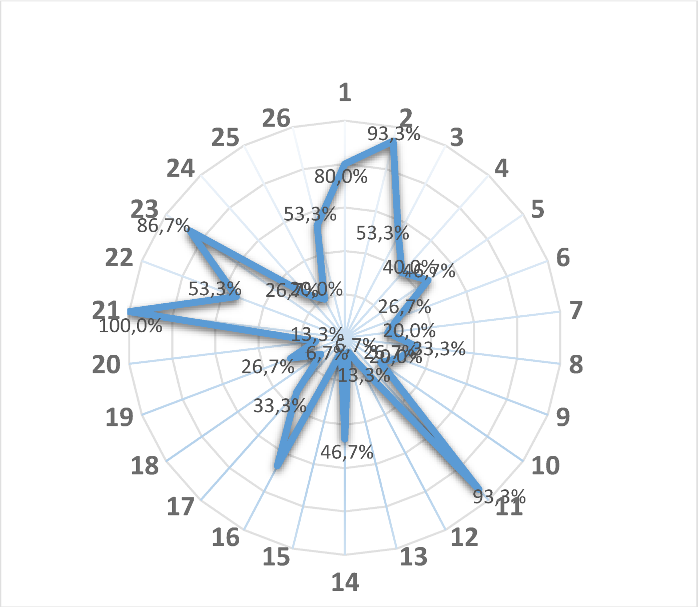

Definition of items: 1. Title/abstract/keywords 2. Introduction.
Methods (items 3-13): 3. Survey details 4. Definitions 5. Outlets 6. Sampling design 7. Samplers 8. Statistical methods 9. Ethical issues 10. Packaging 11. Chemical analysis 12. Method validation 13. Blinding. Results (items 14-20): 14. Outlets (actual) 15. Missing samples 16. Packaging and chemistry results 17. Category of poor quality medicine 18. State company and address as given on packaging 19. Sharing data with MRA 20. Dissemination. Discussion (items 21-24): 21. Key results 22. Limitations 23. Interpretation 24. Intervention. Declaration: 25. Conflict of interest 26. Funding.

No	Item	Penzak, S.R. et al, 2004	M.H.S. Chambuso et al, 2006	WHO, 2007	Musoke. D et al, 2008	Sarr, S. O. et al, 2008	NASCOP, 2012	Chigunta, M.M. et al, 2013
1	Title/abstract/keywords	N	N	Y	Y	Y	N	Y
2	Introduction	Y	Y	Y	Y	Y	Y	Y
**Methods**								
3	Survey details	N	N	Y	Y	N	Y	Y
4	Definitions	N	Y	N	N	N	Y	Y
5	Outlets	N	N	Y	Y	N	N	N

No	Item	Penzak, S.R. et al, 2004	M.H.S. Chambuso et al, 2006	WHO, 2007	Musoke. D et al, 2008	Sarr, S. O. et al, 2008	NASCOP, 2012	Chigunta, M.M. et al, 2013
6	Sampling design	N	N	Y	N	N	N	Y
7	Samplers	N	N	Y	N	N	N	Y
8	Statistical methods	Y	N	N	Y	N	Y	Y
9	Ethical issues	N	N	N	Y	N	N	Y
10	Packaging	N	Y	Y	N	N	N	N
11	Chemical analysis	Y	Y	Y	Y	N	Y	Y
12	Method validation	N	N	Y	N	N	N	N
13	Blinding	N	N	Y	N	N	N	N
**Results**								
14	Outlets	N	N	Y	Y	Y	N	N
15	Missing samples	N	N	N	N	N	N	N
16	Packaging and chemistry results	Y	Y	Y	N	N	N	Y
17	Category of poor-quality medicine	N	N	N	N	N	Y	N
18	State company and address as given on packaging	N	N	N	N	N	N	N
19	Sharing data with MRA	N	N	N	N	Y	Y	N
20	Dissemination	N	N	N	N	N	N	Y
**Discussion**								
21	Key results	Y	Y	Y	Y	Y	Y	Y
22	Limitations	N	N	Y	N	N	Y	Y
23	Interpretation	Y	Y	Y	N	N	Y	Y
24	Intervention	N	N	N	N	Y	Y	N
**Other Information**								
25	Conflict of interest	N	N	N	N	N	N	N
26	Funding	N	N	N	Y	N	Y	N
	Total score	**6**	**7**	**15**	**10**	**6**	**12**	**14**
		**23.1%**	**26.9%**	**57.7%**	**38.5%**	**23.1%**	**46.2%**	**53.8%**

Y : Yes, N : No

No	Item	Krech. L.A et al, 2014	Wang, T et al, 2015	Sapsirisavat , V et al, 2016	Djobet. M.P.N et al, 2017	WHO, 2017	Kamangu, E.N. et al 2017	Wang, X et al, 2019	Mziray S et al, 2021
1	Title/abstract/keywords	Y	Y	Y	Y	Y	Y	Y	Y
2	Introduction	Y	Y	Y	Y	Y	Y	N	Y
**Methods**									
3	Survey details	N	N	Y	Y	Y	Y	N	N
4	Definitions	Y	Y	Y	N	N	N	N	N
5	Outlets	Y	Y	Y	N	N	Y	N	Y
6	Sampling design	N	N	Y	N	Y	N	N	N
7	Samplers	N	Y	N	N	N	N	N	N
8	Statistical methods	N	N	N	Y	N	N	N	N
9	Ethical issues	N	N	N	Y	N	N	N	Y
10	Packaging	N	N	N	Y	N	N	N	N
11	Chemical analysis	Y	Y	Y	Y	Y	Y	Y	Y
12	Method validation	N	N	N	N	N	N	N	Y
13	Blinding	N	N	N	N	N	N	N	N
**Results**									
14	Outlets	N	N	Y	Y	N	N	Y	Y
15	Missing samples	N	N	N	Y	N	N	N	N
16	Packaging and chemistry results	N	Y	Y	Y	Y	Y	N	Y
17	Category of poor-quality medicine	N	N	Y	Y	Y	Y	N	N
18	State company and address as given on packaging	N	Y	N	N	Y	N	N	N
19	Sharing data with MRA	Y	N	N	N	N	N	N	Y
20	Dissemination	N	N	N	N	N	N	N	Y
**Discussion**									
21	Key results	Y	Y	Y	Y	Y	Y	Y	Y
22	Limitations	Y	N	Y	Y	Y	N	Y	N
23	Interpretation	Y	Y	Y	Y	Y	Y	Y	Y
24	Intervention	Y	N	N	N	N	N	N	Y
**Other Information**									

No	Item	Krech. L.A et al, 2014	Wang, T et al, 2015	Sapsirisavat, V et al, 2016	Djobet. M.P.N et al, 2017	WHO, 2017	Kamangu, E.N. et al 2017	Wang, X et al, 2019	Mziray S et al, 2021
25	Conflict of interest	N	N	Y	N	N	N	Y	Y
26	Funding	N	Y	Y	Y	N	Y	Y	Y
	Total score	**10**	**11**	**15**	**15**	**11**	**10**	**8**	**15**
		**38.5%**	**42.3%**	**57.7%**	**57.7%**	**42.3%**	**38.5%**	**30.8%**	**57.7%**

Y : Yes, N : No
