# Supplementary material for: The quality of antiretroviral medicines: an uncertain problem
Source: BMJ Glob Health. 2023 Mar 15;8(3):e011423. doi: 10.1136/bmjgh-2022-011423 (PMC10030546; doi:10.1136/bmjgh-2022-011423)
Supplement: Supplementary data [file bmjgh-2022-011423supp011.pdf]

Supplementary file 11: Description of identified recalls/alerts, seizures and case-reports of substandard and falsified antiretroviral medicines

Reference	Country	Date of incident	Type of publication	Information on the incidents
[1]	Unstated	Jan 1992	Recall/Alert	USFDA urged AIDS buyers clubs to stop the sale and distribution of unauthorized versions of zalcitabine (dideoxycytidine, ddC). The amount of Zalcitabine ranged from no drug to twice the labeled amount. (Buyers clubs are groups that facilitate patient access to drugs purported to be useful for treatment of AIDS or associated conditions).
[2]	Zimbabwe	2001	Case report	A married student aged 33 years from Harare, Zimbabwe, studying in the UK, had started self-treatment with zidovudine tablets bought from a chemist in Zimbabwe. A month later, his CD4 T lymphocyte count and viral load were abnormal. He thus stopped taking the tablets and sent them for analysis. Analysis showed that the tablets contained no zidovudine.
[3]	Tanzania	Aug 2001	Seizure	3.5 tons of re-packaged medicines of various types, including fake preparations for treating AIDS , were seized on 1st of August 2001 in Dar es Salaam. “Two Korean citizens had reportedly done brisk business by selling their AIDS drug, brand named as 'mocrea' and another synthetic compound named 'kissometa'” which they claimed enhanced male potency that were imported illegally into Tanzania.
[4]	USA	2002	Recall/Alert	Falsified labels for Combivir Tablets were placed on two bottles of Ziagen and labels on another two bottles were suspect. Both medicines are used aspart of combination regimens to treat HIV infection.
[5]	Côte d'Ivoire	Nov 2003	Recall/ Alert	The World Health Organization received information about the presence of a falsified triple antiretroviral combination product, Ginovir 3D capsules, in Côte d’Ivoire. According to the Agence Française de Sécurité Sanitaire des Produits de Santé, laboratory investigations of Ginovir 3D samples did not show the presence of lamivudine or indinavir; the capsules were found to contain 201 mg of zidovudine and 40 mg of stavudine per capsule, in addition to an non-identified substance
[6]	USA	Dec 2000	Seizure	In 2000, AmerisourceBergen bought 52 bottles of falsified Retrovir, from a small Ohio wholesaler. The bottles were found during a routine inspection in 2001 at AmerisourceBergen's Orlando distribution center. By turning to the smaller wholesaler rather than buying directly from the drug's manufacturer, AmerisourceBergen saved about $8 per bottle on a product that costs nearly $300 a bottle, sales records showed.
[7]	Democratic Republic of the Congo	2004	Case report	The emergence of falsified antiretroviral drugs in Democratic Republic of Congo has prompted serious concerns among groups that advocate widespread distribution of these drugs in HIV-affected countries. According to Médecins Sans Frontières, fluvoxamine (an antidepressant) and cyclobenzaprine HCl (a

				muscle relaxant) had been labeled as either "Triomune" (a combination of stavudine, lamivudine, and nevirapine) or "Duovir" (a combination of zidovudine and lamivudine), the two commonly prescribed antiretroviral brands that are manufactured by Indian pharmaceutical company Cipla.
[8]	Developing Countries	Nov 2004	Recall/ Alert	Lamivudine 150mg tablet from Cipla Ltd, Kurkumbh, blister pack of 10; and Lamivudine 150mg plus Zidovudine 300mg tablet, Cipla Ltd, Vikhroli, blister pack of 10- The two medicines (which are used in the treatment of AIDS) had been delisted by WHO in May this year due to non-compliance with international standards at the contract research organizations (CROs) hired by Cipla to conduct bioequivalence tests on the products.
[9]	Europe	Jun 2007	Recall/ Alert	The European Medicines Agency (EMEA)issued a Press Release announcing the Europe-wide recall of Viracept (nelfinavir). This recall was initiated after Roche, the manufacturer, identified the presence of ethyl mesylate in some batches of Viracept. Ethyl mesylate is a genotoxic substance that is harmful to DNA.
[10]	USA	Apr 2007	Recall/ Alert	Cases of misbranding cases of two 60-units bottles of Combivir Tablets. Combivir Tablets (in a legitimate bottle) is stated to contain 150 milligrams of lamivudine and 300 milligrams of zidovudine; however, the misbranded bottles of Combivir contained 300 milligram tablets of Ziagen. The falsified labels identified Lot No. 6ZP9760 with expiration dates of April 2010 and April 2009. Company tests have shown no problems with the medicine itself; both Ziagen and Combivir are authentic drug product.
[11]	Germany	2009	Recall/ Alert	In 2009, falsified versions of GlaxoSmithKline's Combivir (lamivudine and zidovudine) and Boehringer Ingelheim's Viramune (nevirapine) were discovered in the legal supply chain in Germany, Combivir in Bremen and Viramune on the island of Sylt in northern Germany.
[12]	UK	2011	Case report	Orifarm, a Danish supplier of parallel-imported and generic pharmaceuticals acknowledged that their supply of Truvada had been compromised by "the presence of counterfeits," reported Securing Pharma. Additionally falsified Viread (tenofovir) has been found in the UK market. MHRA believes that the affected batches contained genuine medication destined for Turkey, however the packaging was falsified. The reason for the substitution of fake packaging is unknown.
[13]	Denmark	Nov 2011	Recall/ Alert	Orifarm said it took the decision to withdraw supplies of Truvada (emtricitabine and tenofovir disoproxil fumarate) - a combination HIV medicine originally developed and marketed by US drugmaker Gilead Sciences, because it has been compromised by the presence of falsified samples.
[14]	Kenya	Sep 2011	Case report	In September 2011, nurses working in an MSF (Médecins Sans Frontières) HIV/AIDS treatment program in Nairobi found two batches of the drug Zidolam-N, a fixed dose combination of zidovudine, lamivudine, and nevirapine, to be molding, friable, and discolored. On close inspection of the suspected products, WHO confirmed that the drugs had been falsified

[15]	Tanzania	Aug 2012	Recall/ Alert	In August, a batch of an unspecified HIV medicine, produced by the Tanzania Pharmaceutical Industry (TPI), was immediately recalled and subjected to further testing, which revealed that it was falsified. 12,000 bottles in the fake ARV batch, 9,570 had so far been successfully recalled.
I[16]	USA	Jul 2012	Seizure	"During an investigation, the FBI seized more than $16 million worth of second-hand prescription drugs, comprised of more than 33,000 bottles and more than 250,000 loose pills, kept in uncontrolled and sometimes egregious conditions by various defendants and their co-conspirators". These were drugs designed to treat various illnesses, including HIV, schizophrenia, and asthma
[17]	UK	2014	Recall/ Alert	"Gilead Sciences Limited is recalling the batches (KFBSD, KFBTD) of Viread 245 mg Film-Coated Tablets due to possible presence of silicone rubber. A failure of equipment used in the manufacture of the active pharmaceutical ingredient formulated in these medicinal product batches may have resulted in damage to silicone gaskets and silicone fragments entering the product in these recalled lots."
[18]	Nigeria	Jan 2014	Recall/ Alert	"The National Agency for Food and Drug Administration and Control, NAFDAC, suspended the use of Tryonex, a brand of Antiretroviral, medicines in the country. This was due to concerns expressed by Treatment Action Movement, TAM, a coalition of HIV activists in Nigeria and other similar organisations."[…] "The package presentation is substandard, the labelling is amateurish, resembling the work of professional counterfeits and street drugs peddlers. Some of the labels were actually upside down with conflicting and incorrect instructions for use." […] "The drug literature, he noted, was also stuffed inside the same plastic pack as the ARVs, giving little assurance of hygienic handling of the tablets during packaging."
[19]	Zimbabwe	Aug 2015	Recall/ Alert	According to Police Commissioner Charity Charamba, from Zimbabwe, 424,000 tablets of fake anti-retroviral drugs were recalled in the country
[20]	Colombia	Nov 2015	Seizure	According to the Colombian authorities, the drugs seized in simultaneous operations on November 22, 2015 in houses located in the cities of San Cristobal, Ciudad Bolivar and Bosa were to be distributed in some 50 pharmacies located in the center, west, and south of Bogota. Fake drugs were treatments against HIV-AIDS . According to the investigation, traffickers had added dyes or iodine in some drugs. In other cases, expired medicines were re-labeled
[21]	France	2016	Recall/Alert	Recall of 2 batches of the specialty VIRAMUNE 400mg, prolonged-release tablet, box of 30cp (lot 559829B -expiry 04/2018; lot 560038A - expiry 06/2018). "This recall follows the discovery of the presence of blisters of a lot in the other lot box"
[22]	Colombia	Jul 2016	Seizure	"On 27 July 2016, Colombian police arrested 10 people suspected of being part of a gang smuggling and counterfeiting medicines. Among the fake drugs, the authorities reported having seized treatments against cancer, HIV-AIDS,

				contraceptives, almost 18 000 units worth 800 million pesos, or about UD$260.000. This network would be located in Bogota, Cucuta and Medellin.”
[23]	Argentina	Jan 2018	Recall/Alert	The National Administration of Drugs, Foods and Medical Devices Argentina (ANMAT) alerted the population on the existence of falsified batches of the following medicinal specialties:<br>Apidra 100 U.I./ml Insulina Glulisina, SoloSta - Lot 5F 964A expired: 10/2018. Kaletra Lopinavir/Ritonavir 200 mg/50mg - Lot 347789D expired 08/2018. Reyataz atazanavir 300mg/30 capsule, lot 4C85179A, expired. ABR 2018.
[24]	Argentina	Jan 2018	Recall/Alert	ANMAT alerted the population on the existence of falsified batches of the following medicinal specialties: ISENTRESS Raltegravir 400 mg, lot ARG0324/L026309. PERJETA Pertuzumab 420 mg/ 14 ml, lot H0109918. VIRORREBER 600 Efavirenz 600 mg, lot MEG35IK4
[25]	Worldwide	Oct 2018	Seizure	“Almost one million packages were inspected during the week of action (9 – 16 October), with 500 tons of illicit pharmaceuticals seized worldwide.These included anti-inflammatory medication, painkillers, erectile dysfunction pills, hypnotic and sedative agents, anabolic steroids, slimming pills and medicines for treating HIV, Parkinson’s and diabetes.”
[26]	Zambia	2016	Case report	“Health Minister, Chitalu Chilufya, reported to have endangered the lives of thousands of patients by purchasing low standard medicines from questionable sources. A report revealed that the Ministry of Health procured expired antiretroviral treatments and laboratory products in 2016.”
[27]	USA	2021	Recall/Alert	The Janssen Pharmaceutical Companies of Johnson & Johnson recently announced that a falsified form of Symtuza (darunavir/cobicistat/emtricitabine/tenofovir alafenamide) has been discovered in three US pharmacies.
[28]	USA	2021	Recall/Alert	“Gilead Sciences became aware of tampered and falsified versions of Biktarvy® (bictegravir 50 mg, emtricitabine 200 mg, and tenofovir alafenamide 25 mg tablets) and Descovy® (emtricitabine 200 mg and tenofovir alafenamide 25 mg tablets) in the US. Distributors not authorized by Gilead to sell Gilead-branded medicine had sold these falsified medicines to pharmacies where genuine Gilead bottles were tampered with a counterfeit foil induction seal or label and contain incorrect tablets”
FDA, Food and Drugs Administration; US, United States; UK, United Kingdom; MHRA, Medicines and Healthcare products Regulatory Agency; HIV-AIDS, Human Immunodeficiency Virus- Acquired Immunodeficiency Syndrome				

1 USFDA. FDA urges buyers’ clubs to stop sales of underground ddC; Roche expands premarketing access programs. Am. J. Hosp. Pharm. 1992;**49**:1024.https://pubmed.ncbi.nlm.nih.gov/1316252/ (accessed 20 Aug 2022).

2 Apoola A, Sriskandabalan PS, Wade AAH. Self-medication with zidovudine that was not [14]. Elsevier B.V. 2001. doi:10.1016/S0140-6736(00)04499-8

3 Panapress. Two Koreans nabbed selling fake AIDS drug. https://drive.google.com/file/d/1sZ9QPbunUtc8bXNyF1l-4n9zQRR1zJH_/view (accessed 5 Sep 2022).

4 USFDA. Combivir (lamivudine plus zidovudine) Press Release, May 2002. 2002.https://drive.google.com/file/d/1ZheYAmAsHNmj58PZ2sld9-9rcu3MDUhr/view (accessed 21 Aug 2022).

5 World Health Organization. Counterfeit triple antiretroviral combination product (Ginovir 3D) detected in Cote d'Ivoire. 2003. https://cdn.who.int/media/docs/default-source/pvg/drug-alerts/da110---drugalert110.pdf?sfvrsn=cacdd476_6 (accessed 21 Aug 2022).

6 Gaul G. U.S. Prescription Drug System Under Attack - The Washington Post. PLoS One. 2003.https://www.washingtonpost.com/archive/politics/2003/10/19/us-prescription-drug-system-under-attack/2ad69f71-42a0-4fce-829d-89fe0d4f3bd3/?… (accessed 21 Aug 2022).

7 Ahmad K. Antidepressants are sold as antiretrovirals in DR Congo. *Lancet* 2004;**363**:713. doi:10.1016/s0140-6736(04)15670-5

8 WHO. Two Cipla AIDS medicines back on WHO's list of prequalified medicines: generic manufacturers continue to upgrade their practices. 2004. https://extranet.who.int/pqweb/news/20041130/two-cipla-aids-medicines-back-whos-list-prequalified-medicines (accessed 21 Aug 2022).

9 WHO. All batches of Viracept (nelfinavir) recalled from the European Market due to possible contamination with a genotoxic substance. 2007. https://drive.google.com/file/d/1f88PZOscaP87FuNpl0XVDA8t3GLheTeU/view (accessed 22 Aug 2022).

10 AIDS Info. Important Notice about Combivir and Ziagen. 2007. https://drive.google.com/file/d/1eERFacn1P7ZGz5LYwAeLprwqkbgXc-Eb/view (accessed 22 Aug 2022).

11 USFDA. Medtronic, Inc. Recalls Dual Chamber Implantable Pulse Generators (IPGs) Due to Possible Circuit Error. 2019.

12 Imber S. Counterfeit HIV Medication Discovered in UK Supply Chain. 2011.https://www.safemedicines.org/2011/12/counterfeit-hiv-medication-discovered-in-uk-supply-chain-391.html (accessed 22 Aug 2022).

13 Taylor P. Counterfeits cause parallel importer to withdraw product in Denmark. https://www.securingindustry.com/pharmaceuticals/counterfeits-cause-parallel-importer-to-withdraw-product-in-denmark/s40/a1120/#.YwMEoHbP0dV (accessed 22 Aug 2022).

14 Cohn J, Von Schoen-Angerer T, Jambert E, *et al.* When falsified medicines enter the supply chain: description of an incident in Kenya and lessons learned for rapid response. *J Public Health Policy* 2013;**34**:22–30. doi:10.1057/JPHP.2012.53

15 Tanzania investigates fake HIV drugs. 2012.https://www.poz.com/article/tanzania-hiv-drugs-23074-8324 (accessed 22 Aug

2022).

16 FBI. Manhattan U.S. Attorney Announces Charges Against 48 Individuals in Massive Medicaid Fraud Scheme Involving the Diversion and Trafficking of Prescription Drugs. 2012.https://archives.fbi.gov/archives/newyork/press-releases/2012/manhattan-u.s.-attorney-announces-charges-against-48-individuals-in-massive-medicaid-fraud-scheme-involving-the-diversion-and-trafficking-of-prescription-drugs (accessed 22 Aug 2022).

17 MHRA. Viread 245 mg film-coated tablets - possible presence of silicone rubber. 2014. https://www.gov.uk/drug-device-alerts/drug-alert-viread-245-mg-film-coated-tablets-possible-presence-of-silicone-rubber (accessed 22 Aug 2022).

18 Tobore O. NAFDAC suspends use of Nigerian government's 'substandard' HIV drug. 2014.https://www.premiumtimesng.com/news/152608-nafdac-suspends-use-nigerian-governments-substandard-hiv-drug.html (accessed 22 Aug 2022).

19 Filactu. Operation GIBOIA II : contre le crime pharmaceutique en Afrique australe. 2015.https://drive.google.com/file/d/1TtCAR9W38gXEQbwXkw54UFqrcs8xcRcg/view (accessed 22 Aug 2022).

20 IRACM. Two tons of counterfeit medicines including treatments against HIV-AIDS seized in Colombia. 2015.https://drive.google.com/file/d/1rFlf3G-7Wf26risQuxc8O1LRkAXu__57/view (accessed 22 Aug 2022).

21 ANSM. Viramune 400 mg prolonged-release tablets (nevirapine) - Boehringer Ingelheim France - Recall of batches. 2016.https://drive.google.com/file/d/1ttu6NhwNokFcSTXPJMGriSNzRrL88vOO/view (accessed 22 Aug 2022).

22 Enforcement: A network of trafficking on fake and counterfeit drugs dismantled in Colombia. 2018.https://drive.google.com/file/d/1y4-BsfEAq7SMov4wRqTKSN_30Y1pEZKa/view (accessed 22 Aug 2022).

23 ANMAT. ANMAT detects counterfeit batches of various medicines. http://www.anmat.gov.ar/comunicados/Apidra-Kaletra-Reyataz_03-01-18.pdf (accessed 22 Aug 2022).

24 ANMAT. ANMAT detects counterfeit batches of various medicines. 2018.http://www.anmat.gov.ar/comunicados/Isentress-Perjeta-Virorreber 06-02-18.pdf (accessed 22 Aug 2022).

25 Interpol. Illicit online pharmaceuticals: 500 tonnes seized in global operation. 2018.https://www.interpol.int/en/News-and-Events/News/2018/Illicit-online-pharmaceuticals-500-tonnes-seized-in-global-operation (accessed 22 Aug 2022).

26 IRACM. Zambia : Health Minister suspected of corruption and procuring fake medicines. 2019.https://drive.google.com/file/d/1OxGKt0FqKx7vQ9XFhh2kj6oOz5iWAPyz/view (accessed 22 Aug 2022).

27 Spinder J. Counterfeit Janssen HIV drug discovered in US pharmacies. 2021.https://www.outsourcing-pharma.com/Article/2021/01/13/Counterfeit-Janssen-HIV-drug-discovered-in-US-pharmacies (accessed 30 Nov 2021).

28 Gilead Warns of Counterfeit HIV Medication Being Distributed in the United States. https://www.gilead.com/news-and-press/company-statements/gilead-warns-of-counterfeit-hiv-medication-being-distributed-in-the-united-states (accessed 24 May 2022).

BMJ Publishing Group Limited (BMJ) disclaims all liability and responsibility arising from any reliance placed on this supplemental material which has been supplied by the author(s)
