# Supplementary material for: The quality of antiretroviral medicines: an uncertain problem
Source: BMJ Glob Health. 2023 Mar 15;8(3):e011423. doi: 10.1136/bmjgh-2022-011423 (PMC10030546; doi:10.1136/bmjgh-2022-011423)
Supplement: Supplementary data [file bmjgh-2022-011423supp012.pdf]

Supplementary file 12: Description of identified recalls/alerts, seizures and case-reports of substandard and falsified antiretroviral related medical devices.

Reference	Country	Date of incident	Type of publication	Information on the incidents
[1]	Kenya	Dec 2011	Recall/Alert	Kenya recalled one million HIV testing kits because of fears about their accuracy. "The WHO had raised an alert about the kit after finding half the test results could be wrong, said Shahnaz Sharif." […]"About 50% of positives may have been reported as negative and 50% of negatives as positive."
[2]	India	2005-2007	Seizure	India seized around 140000 packs of HIV diagnostic test kits. Several cases are described:1/A lot of inconsistencies in the expiry and manufacture dates, number of kits, type of defect (substitution by Pregnancy test or resale of just past-expiry kits), 2/In 2007, a doctor claimed and sue the NACO (National AIDS Control Organization) for not solving the issue, and that some tests were still available. 3/The same doctor raised up a scandal because the kits were procured by the World Bank that tried to hide the issue and found ways to cover them (using CDC agents with link to NACO.)
[3]	France	Aug 2015	Recall/Alert	The blotted membrane unit lot 1412BG006;1501BB001;1501BA001 may generate a higher number of false reactive results than specified on the Instruction for Use.
[4]	Europe	Jun 2016	Recall/Alert	Recall of lots due to possible quality defects in the production process of the raw materials:<br>HIV15100013 (Exp.: 2017-08-31)<br>HIV16010011 (Exp.: 2017-12-31)<br>HIV16020005 (Exp.: 2018-02-28)<br>HCV15100013 (Exp.: 2017-10-31)<br>HCV16010008 (Exp.: 2018-01-31)<br>HCV16030007 (Exp.: 2018-03-31)<br>HBSG15100016 (Exp.: 2017-10-31)<br>HBSG16010012 (Exp.: 2018-01-31)
[5]	UK	Jul 2016	Recall/ Alert	Recall of RightSign HIV 1.2.O Rapid Test Cassette- manufactured by Hangzhou Biotest Biotech Co Ltd: devices may give incorrect results that could lead to a misdiagnosis:<br>HIV14060001<br>HIV14060002<br>HIV14100001<br>HIV14100002<br>HIV15080003<br>HIV15110001<br>HIV16020005<br>HIV16050008
[6]	UK	Sep 2016	Recall/ Alert	"Alert Xpert® HIV-1 Viral Load, Catalog GXHIV-VL-CE-10 lot 1000034821 (cartridge lot 14001) and/or GXHIV-VL-CE-10 lot 1000036280 (cartridge lot 14002) have

				experienced cartridge performance issues that manifest as abnormal PCR amplification curve patterns, which can yield invalid results (the most common outcome), or much less commonly, inaccurate quantification of HIV-1 RNA when using this test."
[7]	Singapore	Jun 2017	Recall/Alert	The Singapore's Health Sciences Authority (HSA) stated that nine lots (SD Biloline HIV Ag/Ab combo kits , all lots from Feb 2016 up to May 2017) of the kits were recalled by the Korea based manufacturer, Standard Diagnostic Inc. due to their reduced sensitivity. According to the manufacturer, when a patient is in the early window period, the lower sensitivity of the affected lots may reduce detection with the possibility of a false negative result for this subset of patients.
[8]	UK	Aug 2017	Recall/ Alert	Recall of Abbott Realtime HIV-1 assay with the lots : 473470;474215;474890,475025,475532,475694,476172,476356,476736,476951,476139 exhibit a higher than expected rate of error codes due to controls out of range or internal control failures, misquantitation, and the potential to not detect HIV
[9]	Guyana	Mar 2020	Recall/ Alert	WHO was informed that at least 8,240 falsified rapid diagnostic tests to detect HIV-1/2 were distributed in Guyana at end-user level. The product was Uni-Gold™ HIV and claimed to be manufactured by Trinity Biotech plc. Genuine lot numbers were HIV7120026 and HIV6120030
[10]	Kenya	Oct 2021	Recall/ Alert	Unapproved HIV self-testing kits in the Kenyan market. There were fears that a number of people could be accessing unapproved and substandard kits in some private health facilities, with possibility of wrong HIV test results.
US, United States; UK, United Kingdom; ; MHRA, Medicines and Healthcare products Regulatory Agency; HIV-AIDS, Human Immunodeficiency Virus-Acquired Immunodeficiency Syndrome; CDC, Centers for Disease Control and Prevention.				

1 Kenya recalls 'faulty' South Korean HIV kits - African Liberty. https://www.africanliberty.org/2011/12/30/kenya-recalls-faulty-south-korean-hiv-kits/ (accessed 29 Aug 2022).

2 Calcutta High Court (Appellete Side) - Unknown vs The State Of West Bengal on 14 November, 2014. 2014. https://indiankanoon.org/doc/22370108/ (accessed 29 Aug 2022).

3 MHRA. Urgent field safety notice -Field Safety correction Action. https://drive.google.com/file/d/1NtO3ObpTDC7MzljwpK213_54JcuF7H6y/view (accessed 16 Sep 2022).

4 MHRA. Urgent Field Safety Notice Field Corrective Action – Recall Diaquick HIV Triline Cassette Diaquick HCV Cassette Diaquick HBSAG Cassette. https://drive.google.com/file/d/1ft3Slpt3tx9LPnV9rpp366lq5Sjk6LA9/view (accessed 16 Sep 2022).

5 MHRA. RightSign HIV 1.2.O Rapid Test Cassette, HCV Rapid Test Cassette & HBsAg Rapid Test Cassette. https://drive.google.com/file/d/1VaATx-qFpOAGfepFFfyGFfRP8brrBqol/view (accessed 16 Sep 2022).

6 MHRA. Urgent Field Safety Notice Xpert® HIV-1 Viral Load. https://drive.google.com/file/d/1wD1mhlqKaLteuhmp7FK-lMcd4RHn6LI3/view (accessed 16 Sep 2022).

7 NAFDAC. Singapore Recalls Faulty HIV Test Kits. 2017.https://www.nafdac.gov.ng/singapore-recalls-faulty-hiv-test-kits/ (accessed 29 Aug 2022).

8 MHRA. Urgent Field Safety Notice Abbott Molecular Inc. https://drive.google.com/file/d/1d6_lqMxmKxj23aY-EbxHQFz1DO50iNND/view (accessed 16 Sep 2022).

9 WHO. Falsified HIV rapid diagnostic test circulating in the WHO regions of the Americas and Africa. Published Online First: 2020.https://www.who.int/medicines/regulation/ssffc/en/ (accessed 29 Aug 2022).

10 Ochieng A. Alarm over ‘fake’ HIV test kits. 2021. https://nation.africa/kenya/news/alarm-over-fake-hiv-test-kits-3577738 (accessed 29 Aug 2022).
