# Supplementary material for: The quality of antiretroviral medicines: an uncertain problem
Source: BMJ Glob Health. 2023 Mar 15;8(3):e011423. doi: 10.1136/bmjgh-2022-011423 (PMC10030546; doi:10.1136/bmjgh-2022-011423)
Supplement: Supplementary data [file bmjgh-2022-011423supp013.pdf]

Supplementary file 13. Other articles on antiretroviral medicines quality included in the review

Title	First author	Year
Determination of Azidothymidine and Its Degradation Product Thymine in Pharmaceutical Dosage Forms by HPLC and HPTLC Densitometry [1]	Tomankova. H	1990
Application of LC-NMR and LC-MS to the identification of degradation products of a protease inhibitor in dosage formulations [2]	Peng, S.X.	1999
Validation of a High-Performance Liquid Chromatography Method for the Assay of and Determination of Related Organic Impurities in Nevirapine Drug Substance [3]	Li Q.Chan	2000
Adequate quality of HIV drugs must be ensured [4]	Arya, S.C.	2002
Comments by Medecins Sans Frontieres to the WHO report [5]	Medecins sans frontieres	2002
Counterfeit Antiretroviral Drugs [6]	Nkengasong, John	2003
AIDS drugs being sold illegally on market stalls in Kenya [7]	Siringi, S.	2004
Hetero Drugs Ltd withdraws Antiretrovirals from WHO prequalification list for further review [8]	WHO	2004
Using Simple Techniques to Detect Poor-Quality ARVs	Phanouvong, S.	2005
Development and validation of a HPLC-UV method for the determination in didanosine tablets [9]	Oliveira, A.M.C.	2005
Counterfeiting of HIV/AIDS Medicines: Implications for Global Epidemic - Recommendations for Workplace Programs [10]	Norris. G B	2005
Dissolution test for lamivudine tablets: Optimization and statistical analysis [11]	Fernandes, C.	2006
Bioavailability study of two oral formulations of didanosine in healthy volunteers [12]	Schramm Andrade, S.	2006
Development and validation of RP-HPLC and ultraviolet spectrophotometric methods of analysis for the quantitative estimation of antiretroviral drugs in pharmaceutical dosage forms [13]	Sarkar, M.	2006
La qualite des medicaments dans les pays les plus defavorises [14]	Videau, J.Y.	2006
Garantir la qualite des medicaments generiques, une demarche encore plus necessaire pour les antiretroviraux	Videau, J.Y.	2006
Etude de la qualite des medicaments generiques DCI achetes par la Pharmacie Populaire du Mali dans le cadre des appels d'offres de 2002 a 2005 [15]	Issiaka C	2006
Stability of ritonavir soft capsule formulation in patients with and without a refrigerator at home in Cote d'Ivoire [16]	Danel, C.	2006
Counterfeit anti-infective drugs [17]	Newton, P.N.	2006
Counterfeit medicines in less developed countries: problems and solutions [18]	Morris. J	2006
Protection Against Counterfeit Pharmaceuticals by Employing GPHF-Minilabs in Health Facilities of Low-income Countries Worldwide [19]	Jahnke. R. W. O.	2006
Determination of 19 antiretroviral agents in pharmaceuticals or suspected products with two methods using high-performance liquid chromatography [20]	Rebiere, H.	2007
Development and validation of a reverse-phase liquid chromatographic method for assay and related substances of abacavir sulfate [21]	Seshachalam, U.	2007
A novel validated LC method for quantitation of lopinavir in bulk drug and pharmaceutical formulation in the presence of its potential impurities and degradation products [22]	Seshachalam, U.	2007
Long-term safety, effectiveness and quality of a generic fixed-dose combination of nevirapine, stavudine and lamivudine [23]	Laurent, C.	2007
Zimbabwe: Fake ARVs Flood Country [24]	-1	2007

Title	First author	Year
Simultaneous reverse phase HPLC estimation of some antiretroviral drugs from tablets [25]	Pai	2007
Mechanisms of prescription drug diversion among drug-involved club- and street-based populations. [26]	Inciardi,JA	2007
Counterfeit or substandard antimicrobial drugs : a review of the scientific evidence [27]	Kelesidis. T	2007
Dangerous medicines: Unproven AIDS cures and counterfeit antiretroviral drugs [28]	Amon, J.J.	2008
Problems associated with substandard and counterfeit drugs in developing countries: a review article on global implications of counterfeit drugs in the era of anti-retroviral (ARVs) drugs in a free market economy [29]	Nsimba, S.E.D.	2008
Quality Control of Protease Inhibitors [30]	Yekkala, R.	2008
Impurity profile study of lopinavir and validation of HPLC method for the determination of related substances in lopinavir drug substance [31]	Chitturi, S.R.	2008
A Simple and Rapid RP-HPLC Method for the Estimation of Nevirapine in Bulk and Pharmaceutical Dosage Forms [32]	Mohanraj. P	2008
Steady-state pharmacokinetic comparison of generic and branded formulations of stavudine, lamivudine and nevirapine in HIV-infected Ugandan adults [33]	P B Kibwika	2008
Determination of lamivudine and stavudine in pharmaceutical preparations using chemometrics-assisted spectrophotometry [34]	Mohamed, A.M.I.	2009
Bioequivalence Study of Two Fixed Dose Combination Tablet Formulations of Lopinavir and Ritonavir in Healthy Volunteers [35]	Chachad, S.	2009
Confusion over counterfeit drugs in Uganda. [36]	Anderson, T	2009
Counterfeit Drugs: Coming to a Pharmacy Near You with an Update for 2009 [37]	Yankus. W	2009
Method development and validation for the estimation of atazanavir in bulk and pharmaceutical dosage forms and its stress degradation studies using UV-VIS spectrophotometric method [38]	Dey, S.	2010
Strengthening National Capacity in Medicines Quality: Five-year Summary (2005-2009)	Phanouvong, S.	2010
Simple and Rapid RP-HPLC Method to Determine the Purity of the Antiretroviral Drug Lamivudine [39]	Kakkar. S	2010
Comparison of HPLC & spectrophotometric methods for estimation of antiretroviral drug content in pharmaceutical products [40]	Kumar. H.A.K	2010
Simultaneous estimation of Zidovudine and Lamivudine tablets by RP-HPLC method	Venkatesh, P.	2011
Simultaneous Determination of Antiretroviral Zidovudine, Lamivudine and Efavirenz by RP HPLC-DAD	Soares, M.F.L.R.	2011
Gradient RP-HPLC method for the determination of potential impurities in atazanavir sulfate	Chitturi. S.R	2011
Quantitative Analysis of Tenofovir by Titrimetric, Extractive Ion-pair Spectophotometric and Charge- Transfer Complexation Methods	Onah J.O	2011
Government warns of fake HIV/Aids drugs	Kasasira. R	2011
Mylan declares to expanding access in developing countries to high quality, affordable HIV/AIDS treatment	Pharmabiz	2011
Development and validation of UV spectroscopic method for determination of atazanavir sulphate in bulk and formulation	Konidala, S.K.	2012
HPTLC methods to assay active ingredients in pharmaceutical formulations: A review of the method development and validation steps	Shewiyo. D	2012
Spectrophotometric Determination of Zidovudine-Loaded Microemulsion and Application in Assay of In Vitro Release Kinetics	Andre L.M.C	2012
Toward Global Standards for Comparator Pharmaceutical Products: Case Studies of Amoxicillin, Metronidazole, and Zidovudine in the Americas	Raimar Lobenberg	2012

Title	First author	Year
Substandard and counterfeit antimicrobials: recent trends and implications to key public health interventions in developing countries	Tadeg, H.	2012
Method development and validation for the estimation of Adefovir dipivoxil in bulk and pharmaceutical dosage forms and its stress degradation studies using UV-VIS spectrophotometric method	Bhirud, C.H.	2013
Counterfeit drugs flood Zim market	-1	2013
Review of the Quality of Pediatric Medications in Developing Countries	Conway, J.	2013
Determination of Zalcitabine in Medicaments by Differential Pulse Voltammetry	Leandro. K C	2013
Falsified lamivudine/zidovudine/nevirapine tablets: rapid identification using X-ray fluorescence technique	Unknown	2013
Antiretroviral medication diversion among HIV-positive substance abusers in South Florida.	Surratt HL	2013
Pretreatment antiretroviral exposure from recreational use.	Grelotti,DJ	2013
Analytical Method Development and Validation by RP-HPLC for the Simultaneous Estimation of Abacavir Sulphate and Lamivudine in Tablet Dosage Forms	Suri, D.	2013
Counterfeit drugs and medical devices in developing countries	Glass, B.D.	2014
Black Market HIV/AIDS Drugs in the News, 2006 – 2013	Safe Medicines	2014
UV spectrophotometric method development and validation for the quantitative estimation of indinavir sulfate in capsules	Rathod, B.H.	2014
Recent Applications of Analytical techniques for counterfeit drug analysis: A Review	Kumar, R.	2014
Recent research on HPLC method of analysis of Lamivudine and Zidovudine: A review	Chowdary K. P. R.	2014
Vulnerable infected populations and street markets for ARVs: Potential implications for PrEP rollout in the USA.	Kurtz,SP	2014
Development and validation of a stability-indicating RP – HPLC method for estimation ofatazanavir sulfate in bulk	Dey, S.	2015
Development and validation of UV spectrophotometric method for estimation of Dolutegravir sodium in tablet dosage form	Balasaheb, B.G.	2015
Simultanious determination of antiretroviral drugs emtricitabine and tenofovir desoproxil fumarate by a stability indicating RP-HPLC method	Valli Purnima, B.	2015
Development and in-vitro characterization of abacavir and zidovudine tablet in combination	Martha, S.	2015
Monitoring the Quality of Medicines: Results from Africa, Asia, and South America	Hajjou, M.	2015
High performance liquid chromatography with PDA detector for combined determination of emtricitabine, tenofovir and efavirenz	Dubey, S.S.	2015
Substandard/Counterfeit Antimicrobial Drugs	Kelesidis.T	2015
Identification, synthesis and characterization of new impurities in tenofovir	He. J	2015
A Simultaneous Determination of Related Substances by High Performance Liquid Chromatography in a Drug Product Using Quality by Design Approach	Tol. T	2015
Counterfeit drugs: Problem of developing and developed countries	Yadav. S	2015
The Demand for Antiretroviral Drugs in the Illicit Marketplace: Implications for HIV Disease Management Among Vulnerable Populations.	Tsuyuki,K	2015
Malaria, tuberculosis: the threat of fake drugs	Cordonnier, C.	2015
Development and validation of RP-HPLC method for simultaneous estimation of Lamivudine and zidovudine in bulk	Kamala, G.	2016

Title	First author	Year
Develoment and validation of UV spectroscopic method for the determination of didanosine in pharmaceutical dosage forms	Ahmed, M.S.M	2016
Simultaneous estimation of lamivudine and stavudine by RP-HPLC in tablet dosage form	Gunasheela, D.	2016
Spectrophotometric analytical method development and validation for simultaneous estimation of a lamivudine and stavudine in solid dosag form	Ratan, K.	2016
Analytical method development and validation for simultaneous estimation of lamivudine and zidovudine in solid dosage form by RP-HPLC method	Sonawane, S. L	2016
Development and Validation of UV Spectroscopic Method for the determination of didanosine in pharmaceutical dosage forms	Ahmed, M	2016
A New and Validated Stability Indicating RP-HPLC Analysis of Darunavir and Cobicistat in Bulk Drug and Tablet Dosage Form	Rizwan, S.H.	2016
Development and Validation of Stability Indicating Method for Darunavir with Forced Degradation Studies Using LC-ESI-MS/MS	Ghante, M.	2016
Development of High Performance Thin Layer Chromatography for Simultaneous Analysis of Lamivudine and Tenofovir Disoproxil Fumarate	Bizimana, T.	2016
Determination of Darunavir and Cobicistat Simultaneously Using Stability Indicating RP-HPLC Method	Nalini, M.V.S.S.	2016
UV-visible spectrophotometric method for the determination of lamivudine in pharmaceutical formulations and human blood samples with MBTH	Reddy, K.S.	2016
Assay and stability studies of cobicistat and atazanavir sulphate in combined dosage form by RP-UPLC method	Purnima V.B	2016
Simultaneous Quantification of Novel Antiretroviral Drug Combination by Stability-indicating High Performance Liquid Chromatography Method	Rao, N. M.	2016
Synthesis and characterization of Tenofovir disoproxil fumarate impurities, anti HIV drug substance	Varal. D	2016
Characterization of drug authenticity using thin-layer chromatography imaging with a mobile phone	Yu, H.	2016
Alere's rapid point-of-care fourth-generation test, HIV combo test receives WHO prequalification	Pharmabiz	2016
Policy makers' inaction is leading people to take PrEP 'in the wild'	Pegoby,R	2016
Development and Validation of HPTLC Method for The Estimation of Antiretroviral Drugs and Their Pharmaceutical Formulations	Joshi, C. N.	2016
Poor-Quality and Counterfeit Drugs: A Systematic Assessment of Prevalence and Risks Based on Data Published From 2007 to 2016.	Koczwara, A.	2017
Simultaneous determination of newly developed antiviral agents in pharmaceutical formulations by HPLC-DAD	Al-Zoman, N.Z.	2017
Simultaneous Determination of Impurities of Atazanavir and Ritonavir in Tablet Dosage Form by Using Reversed-Phase Ultra Performance Liquid Chromatographic Method	Mantripragada. M.k V V N	2017
Identification of degradation products of saquinavir mesylate by ultra-high-performance liquid chromatography/electrospray ionization quadrupole time-of-flight tandem mass spectrometry and its application to quality control.	Thummar. M	2017
Application of Design Space Optimization Strategy to The Development of LC Method for Simultaneous Analysis of 18 Antiretroviral Medicines and 4 Major Excipients Used in Various Pharmaceutical Formulations	Habyalimana. V	2017
Development and Validation of Stability-indicating HPLC method for simultaneous estimation of Tenofovir, Emtricitabine and Efavirenz in fixed dose combination drug product	Sreelatha. P	2017
Development and Validation for the Simultaneous Estimation of Lamivudine, Tenofovir Disproxil and Dolutegravir In Drug Product by RP-HPLC	Nekkala. K	2017
Formulation Development and in vivo Evaluation of Nevirapine Solid Dispersions by Solvent Evaporation Technique	Viswaja. M	2018

Title	First author	Year
Development and Validation of First Order Derivative Method for Tenofovir alafenamide in Bulk using UV Visible Spectroscopy	Dudhe P.B	2018
Development and Validation of RP-HPLC Method for the Simultaneous Estimation of Lamivudine, Tenofovir Alafenamide and Dolutegravir Bulk and their Combined Dosage Form	Mastanamma. Sk	2018
A new quantitative reverse phase high-performance liquid chromatographic method for the quantification of Rilpivirine hydrochloride in bulk and dosage form	Patel. S	2018
Development of a UV-spectrophotometric method for study of degradation profile of tenofovir alafenamide	M. P. Shinde	2018
Antimicrobial Resistance and Substandard and Falsified Medicines: The Case of HIV/AIDS	Suthar A.B	2018
Analytical Method Development and Validation for the Simultaneous Estimation of Abacavir and Lamivudine by Reversed-phase High performance Liquid Chromatography in Bulk and Tablet Dosage Forms	Ahmad. S.A.R	2018
Determination of Simultaneous Estimation HPLC Method for Elvitegravir, Tenofovir Disoproxil Fumarate, Emtricitabine and Cobicistat it’s Pure And Tablet Form	Godasu. S.K	2018
Development and Validation of HPLC Method for Simultaneous Estimation of Emtricitabine, Rilpivirine and Tenofovir Disoproxil Fumarate Tablet Dosage form	Karunakranth. D	2018
RP-HPLC Method for Simultaneous Determination of Lamivudine, Zidovudines and Nevirapine from Their Combined Tablet Dosage Form	Shamili	2018
Rogue pharmacies switch tactics as enforcement bites	Taylor, P.	2018
Development and validation of HPLC methods for simultaneous analysis of 6 antiretrovirals in pharmaceutical formulations	Jocelyn. M. K	2019
Falsified antimicrobial medicine: A neglected global health crisis contributing to drug resistance	Slade, E	2019
Les medicaments falsifies	Hugo RICCI	2019
Falsified and substandard drugs: Stopping the pandemic	Nayyar, GML	2019
La confusion entre "mauvaise qualite" et generiques est nefaste	Favereau, Eric	2019
Method Development, Validation and Forced Degradation Studies of Ritonavir, An Retroviral Drug Using UV-Visible Spectroscopy	K. Bhavyasri	2019
Development and validation of a high performance liquid chromatography method to determine nevirapine in plasma in a resource-limited setting	Faithful M-C	2019
Colombie : demantelement d’un trafic de faux medicaments contre le cancer et le sida	Unknown	2019
Drug polymorphism identification using Fourier Transform-Raman spectroscopy: A comparative study of lamivudine and finasteride drugs	Rao, CS	2021
The epidemic of substandard and falsified medications in Iraq: Evaluating the effectiveness of National Pharmacovigilance Alerts to Community Pharmacies	Al-Jumaili, AZ	2021

1 Tomankova H, Šabartová J. Determination of azidothymidine and its degradation product thymine in pharmaceutical dosage forms by HPLC and HPTLC densitometry. *Chromatogr 1990 291* 1990;**29**:44–50. doi:10.1007/BF02261138

2 Peng SX, Borah B, Dobson RLM, *et al.* Application of LC-NMR and LC-MS to the identification of degradation products of a protease inhibitor in dosage formulations. *J Pharm Biomed Anal* 1999;**20**:75–89. doi:10.1016/S0731-7085(98)00311-2

3 Li QC, Tougas T, Cohen K, *et al.* Validation of a high-performance liquid chromatography method for the assay of and determination of related organic impurities in nevirapine drug substance. *J Chromatogr Sci* 2000;**38**:246–54. doi:10.1093/CHROMSCI/38.6.246

4 Arya SC. Adequate quality of HIV drugs must be ensured. *BMJ Br Med J* 2002;**324**:235. doi:10.1136/BMJ.324.7331.235B

5 Médecins Sans Frontières Access Campaign. Comments by Médecins Sans Frontières to the WHO report. 2002. https://msfaccess.org/comments-medecins-sans-frontieres-who-report (accessed 29 Aug 2022).

6 Nkengasong J. Counterfeit Antiretroviral Drugs. 2003. https://www.medscape.com/viewarticle/465033 (accessed 29 Aug 2022).

7 Siringi S. AIDS drugs being sold illegally on market stalls in Kenya. *Lancet (London, England)* 2004;**363**:377. doi:10.1016/S0140-6736(04)15479-2

8 WHO. Hetero Drugs Ltd withdraws antiretrovirals from WHO prequalification list for further review. *WHO* 2004.

9 Cavalcanti De Oliveira AM, Raposo Löwen TC, Cabral LM, *et al.* Development and validation of a HPLC-UV method for the determination in didanosine tablets. *J Pharm Biomed Anal* 2005;**38**:751–6. doi:10.1016/J.JPBA.2005.02.004

10 Norris GB. Counterfeiting of HIV/AIDS medicines : implications for global epidemic : recommendations for workplace programs. Published Online First: 2005.https://scholar.sun.ac.za:443/handle/10019.1/50307 (accessed 29 Aug 2022).

11 Fernandes C, Junqueira RG, Campos LMM, *et al.* Dissolution test for lamivudine tablets: optimization and statistical analysis. *J Pharm Biomed Anal* 2006;**42**:601–6. doi:10.1016/J.JPBA.2006.05.009

12 Andrade SS, Kano EK, De Lima Souza Brioschi TM, *et al.* Bioavailability study of two oral formulations of didanosine in healthy volunteers. *Arzneimittelforschung* 2006;**56**:359–64. doi:10.1055/S-0031-1296734

13 Sarkar M, Khandavilli S, Panchagnula R. Development and validation of RP-HPLC and ultraviolet spectrophotometric methods of analysis for the quantitative estimation of antiretroviral drugs in pharmaceutical dosage forms. *J Chromatogr B Analyt Technol Biomed Life Sci* 2006;**830**:349–54. doi:10.1016/J.JCHROMB.2005.11.014

14 Videau J. La qualité des médicaments dans les pays les plus défavorisés. *undefined* 2006.

15 C I. Étude de la qualité des médicaments génériques DCI achetés par la Pharmacie Populaire du Mali dans le cadre des appels d offres de 2002 à 2005. Published Online First: 2006.https://docplayer.fr/56441230-Etude-de-la-qualite-des-medicaments-generiques-dci-achetes-par-la-pharmacie-populaire-du-mali-dans-le-cadre-des-appels-d-offres-de-2002-a-2005.html (accessed 29 Aug 2022).

16 Danel C, Peytavin G, Moh R, *et al.* Stability of ritonavir soft capsule formulation in patients with and without a refrigerator at home in Côte d'Ivoire. *Int J STD AIDS* 2006;**17**:784–5. doi:10.1258/095646206778691040

17 Newton PN, Green MD, Fernández FM, *et al.* Counterfeit anti-infective drugs. *Lancet Infect Dis* 2006;**6**:602–13. doi:10.1016/S1473-3099(06)70581-3

18 Julian Morris PS. *Counterfeit medicines in less developed countries : problems and solutions*. 2006. https://www.worldcat.org/title/counterfeit-medicines-in-less-developed-countries-problems-and-solutions/oclc/69017922 (accessed 29 Aug 2022).

19 O. JRW. Protection against counterfeit pharmaceuticals by employing GPHF-minilabs in health facilities of low-income countries worldwide. 2006. https://www.researchgate.net/publication/290227581_Protection_against_counterfeit_pharmaceuticals_by_employing_GPHF-minilabs_in_health_facilities_of_low-income_countries_worldwide (accessed 29 Aug 2022).

20 Rebiere H, Mazel B, Civade C, *et al.* Determination of 19 antiretroviral agents in pharmaceuticals or suspected products with two methods using high-performance liquid chromatography. *J Chromatogr B* 2007;**850**:376–83. doi:10.1016/J.JCHROMB.2006.12.007

21 Seshachalam U, Haribabu B, Chandrasekhar KB. Development and validation of a reverse-phase liquid chromatographic method for assay and related substances of abacavir sulfate. *J Sep Sci* 2007;**30**:28–34. doi:10.1002/JSSC.200600209

22 Seshachalam U, Haribabu B, Chandrasekhar KB. A novel validated LC method for quantitation of lopinavir in bulk drug and pharmaceutical formulation in the presence of its potential impurities and degradation products. *Biomed Chromatogr* 2007;**21**:716–23. doi:10.1002/BMC.810

23 Laurent C, Kouanfack C, Koulla-Shiro S, *et al.* Long-term safety, effectiveness and quality of a generic fixed-dose combination of nevirapine, stavudine and lamivudine. *AIDS* 2007;**21**:768–71. doi:10.1097/QAD.0B013E328045C4D7

24 Zimbabwe: Fake ARVs Flood Country - allAfrica.com. 2007.https://allafrica.com/stories/200707260858.html (accessed 29 Aug 2022).

25 Pai N, Desai AD. Simultaneous reverse phase HPLC estimation of some antiretroviral drugs from tablets. *Indian J Pharm Sci* 2007;**69**:118–20. doi:10.4103/0250-474X.32123

26 Inciardi JA, Surratt HL, Kurtz SP, *et al.* Mechanisms of prescription drug diversion among drug-involved club- and street-based populations. *Pain Med* 2007;**8**:171–83. doi:10.1111/J.1526-4637.2006.00255.X

27 Kelesidis T, Kelesidis I, Rafailidis PI, *et al.* Counterfeit or substandard antimicrobial drugs: a review of the scientific evidence. *J Antimicrob Chemother* 2007;**60**:214–36. doi:10.1093/JAC/DKM109

28 Amon JJ. Dangerous medicines: Unproven AIDS cures and counterfeit antiretroviral drugs. *Global Health* 2008;**4**:5. doi:10.1186/1744-8603-4-5

29 Nsimba SED. Problems associated with substandard and counterfeit drugs in developing countries: a review article on global implications of counterfeit drugs in the era of antiretroviral (ARVs) drugs in a free market economy. *East Afr J Public Health* 2008;**5**:205–10. doi:10.4314/EAJPH.V5I3.39004

30 Yekkala R, Adams E, Van Schepdael A, *et al.* Quality control of protease inhibitors. *J Pharm Sci* 2008;**97**:2012–21. doi:10.1002/jps.21137

31 Chitturi SR, Bharathi C, Reddy AVR, *et al.* Impurity profile study of lopinavir and validation of HPLC method for the determination of related substances in lopinavir drug substance. *J Pharm Biomed Anal* 2008;**48**:1430–40. doi:10.1016/J.JPBA.2008.09.015

32 Mohanraj P, Sarkar DK, Choudhury T, *et al.* A simple and rapid RP-HPLC method for the estimation of nevirapine in bulk and pharmaceutical dosage forms. *E-Journal Chem* 2008;**5**:1081–6. doi:10.1155/2008/358654

33 Byakika-Kibwika P, Lamorde M, Kalemeera F, *et al.* Steady-state pharmacokinetic comparison of generic and branded formulations of stavudine, lamivudine and nevirapine in HIV-infected Ugandan adults. *J Antimicrob Chemother* 2008;**62**:1113–7. doi:10.1093/JAC/DKN290

34 Mohamed AEMI, Mikre W. Determination of lamivudine and stavudine in pharmaceutical preparations using chemometrics-assisted spectrophotometry. *Saudi Pharm J* 2009;**17**:275–81. doi:10.1016/J.JSPS.2009.10.003

35 Chachad S, Lulla A, Malhotra G, *et al.* Bioequivalence study of two fixed dose combination tablet formulations of lopinavir and ritonavir in healthy volunteers. *Arzneimittelforschung* 2009;**59**:263–8. doi:10.1055/S-0031-1296395

36 Anderson T. Confusion over counterfeit drugs in Uganda. *Lancet (London, England)* 2009;**373**:2097–8. doi:10.1016/S0140-6736(09)61134-X

37 Yankus W. Counterfeit Drugs: Coming to a Pharmacy Near You with an Update for 2009. In: *The American Council on Science and Health*. 2009. https://www.acsh.org/wp-content/uploads/2012/04/20090202_counterfeitdrug09.pdf (accessed 5 Sep 2022).

38 Dey S, Reddy YV, Reddy T, *et al.* Method development and validation for the estimation of atazanavir in bulk and pharmaceutical dosage forms and its stress degradation studies using UV-VIS spectrophotometric method | Request PDF. *Int J Pharma Bio Sci* Published Online First: 2010.https://www.researchgate.net/publication/286890638_Method_development_and_validation_for_the_estimation_of_atazanavir_in_bulk_and_pharmaceutical_dosage_forms_and_its_stress_degradation_studies_using_UV-VIS_spectrophotometric_method (accessed 5 Sep 2022).

39 Kakkar S, Mohanraj K, Deep A, *et al.* Simple and Rapid RP-HPLC Method to Determine the Purity of the Anti-retroviral Drug Lamivudine. *Lat Am J Pharm*

Published Online First: 2010.https://www.researchgate.net/publication/236644939_Simple_and_Rapid_RP-HPLC_Method_to_Determine_the_Purity_of_the_Anti-retroviral_Drug_Lamivudine (accessed 5 Sep 2022).

40 Kumar AKH, Sudha V, Swaminathan S, *et al.* Comparison of HPLC & spectrophotometric methods for estimation of antiretroviral drug content in pharmaceutical products. *Indian J Med Res* Published Online First: 2010.https://pubmed.ncbi.nlm.nih.gov/20966516/ (accessed 5 Sep 2022).
